# Honeybee locomotion is impaired by Am-Ca_V_3 low voltage-activated Ca^2+^ channel antagonist

**DOI:** 10.1038/srep41782

**Published:** 2017-02-01

**Authors:** M. Rousset, C. Collet, T. Cens, F. Bastin, V. Raymond, I. Massou, C. Menard, J.-B. Thibaud, M. Charreton, M. Vignes, M. Chahine, J. C. Sandoz, P. Charnet

**Affiliations:** 1 Institut des Biomolécules Max Mousseron (IBMM), UMR 5247, CNRS ENSCM, Université de Montpellier, bât. CRBM, 1919 Route de Mende, 34293 Montpellier cedex 5, France; 2 INRA, UR 406 Abeilles et Environnement, 228 Route de l’aérodrome, Domaine Saint Paul, Site Agroparc, CS40509, 84914 Avignon cedex 9, France; 3 Evolution Génomes Comportement et Ecologie, CNRS, Univ. Paris-Sud, IRD, Université Paris Saclay, 1 avenue de la Terrasse, F-91198 Gif-sur-Yvette, France; 4 RCIM, UPRES-EA2647 USC INRA 1330, SFR QUASAV 4207, Université d’Angers, 2 Bld Lavoisier, 49045 ANGERS Cedex 01, France; 5 CRCA, UMR 5169, CNRS, Université Paul Sabatier, Bâtiment IVR3, 118 route de Narbonne, 31062 Toulouse, France; 6 Centre de recherche, Institut universitaire en santé mentale de Québec, 2601 Chemin de la Canardière, Québec (Québec), G1J 2G3, Canada

## Abstract

Voltage‐gated Ca^2+^ channels are key transducers of cellular excitability and participate in several crucial physiological responses. In vertebrates, 10 Ca^2+^ channel genes, grouped in 3 families (*Ca*_*V*_*1, Ca*_*V*_*2* and *Ca*_*V*_*3*), have been described and characterized. Insects possess only one member of each family. These genes have been isolated in a limited number of species and very few have been characterized although, in addition to their crucial role, they may represent a collateral target for neurotoxic insecticides. We have isolated the 3 genes coding for the 3 Ca^2+^ channels expressed in *Apis mellifera*. This work provides the first detailed characterization of the honeybee T-type Ca_V_3 Ca^2+^ channel and demonstrates the low toxicity of inhibiting this channel. Comparing Ca^2+^ currents recorded in bee neurons and myocytes with Ca^2+^ currents recorded in Xenopus oocytes expressing the honeybee *Ca*_*V*_*3* gene suggests native expression in bee muscle cells only. High‐voltage activated Ca^2+^ channels could be recorded in the somata of different cultured bee neurons. These functional data were confirmed by *in situ* hybridization, immunolocalization and *in vivo* analysis of the effects of a Ca_V_3 inhibitor. The biophysical and pharmacological characterization and the tissue distribution of Ca_V_3 suggest a role in honeybee muscle function.

Locomotion, olfactory learning and memory are key processes by which honeybees can forage, detect food, individuals and hives. These processes are crucial and their impairment could compromise the survival of the whole colony. However, while olfaction, learning or memory have been described and studied extensively over the past decades^1^, some of their molecular actors are still poorly characterized. Ca^2+^ sensitive dyes and electrophysiological studies on olfactory neurons^2^, antennal lobes neurons, projection neurons^3^,^4^, Kenyons cells^5^,^6^, or muscle cells^7^ revealed that the Ca^2+^ influx plays a major role^8^, but produced neither a clear molecular identification and spatial expression pattern of the Ca^2+^ channels nor a precise description of their role in odor processing or locomotion. This is all the more regrettable since the voltage-gated Ca^2+^ channels have been shown to be a secondary target of pyrethroids^9^, a widely used class of neurotoxic insecticides targeting Na^+^ channels^10^.

A careful analysis of the Ca_V_ channels expressed in insects^11^,^12^ suggests that multiple Ca^2+^ channel types – including low- and high-voltage activated (LVA and HVA, respectively) – are present and differentially expressed^11^,^12^. While HVA Ca^2+^ channels are routinely described^4^,^7^,^13^, no T type-like/LVA/type 3 Ca^2+^ channels have been reported yet in honeybees despite the fact that a *Ca*_*V*_*3* gene has been identified and isolated^12^. In *Drosophila*, a *Ca*_*V*_*3* gene has been recently identified, cloned, and characterized^14^ but its invalidation did not lead to any obvious phenotype and its physiological role remains unclear. Very few examples of T-type currents have been described in other insects^3^,^15^,^16^,^17^. In the absence of a precise pharmacological profile for this channel, a detailed analysis of its role in honeybee physiology remains challenging, though this role may be of importance due to the lack of sodium currents in insect muscles^7^,^18^,^19^. Our recent identification of the honeybee genes *Am-*Ca_V_1, *Am-*Ca_V_2 and *Am-*Ca_V_3 encoding the three types of voltage-gated Ca^2+^ channels^12^,^20^ gives us the opportunity to perform a detailed biophysical characterization of the bee LVA channel and to analyze its role in the generation of Ca^2+^ currents recorded in honeybee neurons and muscle cells.

Here, we demonstrated, using functional expression in Xenopus oocytes, that *Am-*Ca_V_3 does encode a low-threshold T-type Ca_V_ channel with an unusual pharmacological profile, being blocked by mibefradil but not by amiloride, TTa-A2, or the insect-specific toxin Atx-2. *Am-*Ca_V_3 expression was tissue specific with a lack of T-type Ca^2+^ currents in several honeybee neuron types of the olfactory pathway, but a clear expression in muscle cells. Mibefradil, used *in vivo*, led to locomotion deficits but to a mild perturbation of olfactory learning. Altogether, these data indicate that the Ca_V_3 channel plays a putative role in honeybee muscle function and suggest that pharmacology of bee HVA Ca^2+^ channels should now be documented to help future analysis of their roles in bee physiology

## Results

### Functional and pharmacological characterization of Am-Ca_V_3 expressed in X. oocytes

**Figure 1:**
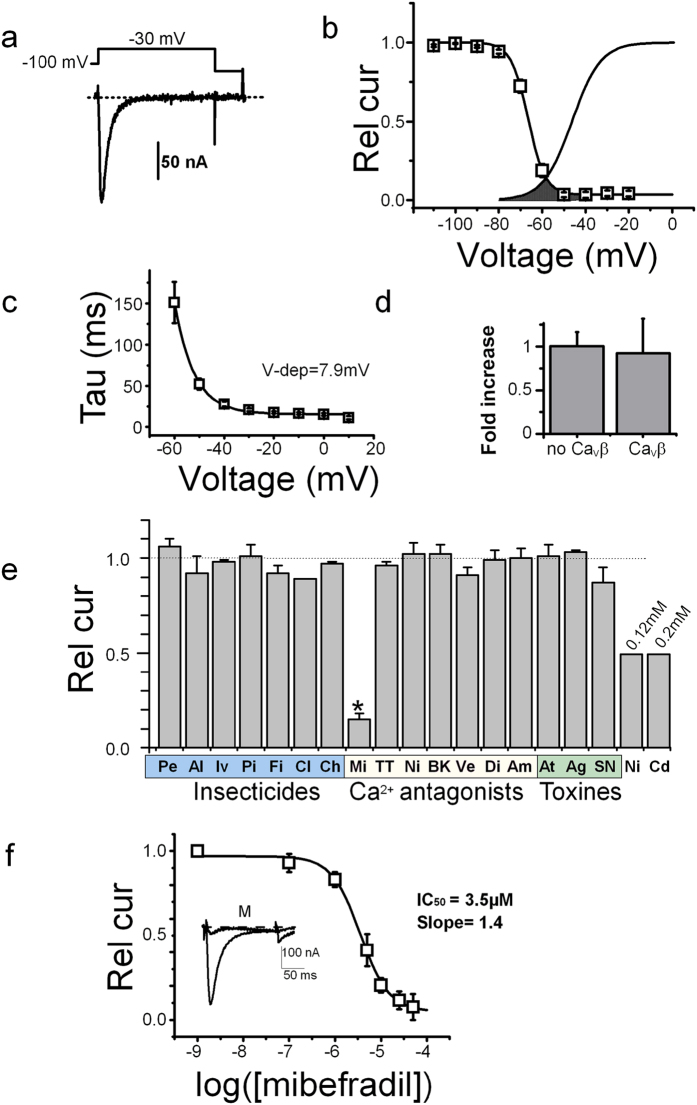
Functional characterization of Am-Ca_V_3 in Xenopus oocytes. (**a**) Ba^2+^ Current traces recorded from oocytes expressing *Am*-Ca_V_3. Holding potential: −100 mV, Step potential −30 mV, duration: 150 ms. (**b**) Averaged activation and inactivation curves recorded from *Am*-Ca_V_3-injected oocytes. The inactivation curves were obtained using 2.5 s conditioning potentials from −100 to various potentials (10 mV increment) followed by a subsequent test pulse to −30 mV. Voltages for half-activation (V_act_): −47 ± 1 mV, activation slope: 6.7 ± 0.3 mV, voltage for half-inactivation (V_inact_): −66 ± 1 mV, inactivation slope: 3.5 ± 0.2 mV, percentage of non-inactivating current: 5 ± 1% (n = 12). The window current, is underlined as a dotted surface. (**c**) Current inactivation time-course was fitted at each potential using a mono-exponential decay with a time constant Tau. The voltage-dependence of Tau displayed an exponential decrease with an e-fold decrease (Vdep.) every 7.9 ± 0.4 mV (n = 10). (**d**) Increase in Ba^2+^ current amplitudes recorded on oocytes expressing Ca_V_3 Ca^2+^ channel produced by the coexpression of the Ca_V_β subunit^20^. Currents were normalized to the current amplitude of *Am*-Ca_V_3 without Ca_V_β (n = 4). (**e**) Pharmacological profile of Am- Ca_V_3 Ca^2+^ channels defined by the response to several calcium antagonists, insecticides and toxins. The residual currents in response to these antagonists are shown relative to the current recorded in control condition. Pe: permethrin 50 μM; Al: allethrin 50 μM: Iv: ivermectine 10 μM; Pi: picrotoxin 10 μM; Fi: fipronil 10 μM; Cl: clothianidine 10 μM; Ch: Chlorantraniliprole 10 μM; Mi: mibefradil 10 μM; NN: NNC 55-0396 10 μM; TT: TTA-A2 10 μM; Ni: nifedipine: 10 μM; BK: bay-k 8644 10 μM; Ve: verapamil 10 μM; Di: diltiazem 10 μM; Am: amiloride 1 mM; At: atrachotoxin 10 μM; Ag: w-aga-IV-A 10 μM; SN: SNX 482 10 μM; Ni: nickel chloride 0.12 mM, CdCl_2_: cadmium chloride 0.2 mM. (**f**) Dose-response curve of mibefradil on *Am*-Ca_V_3 current amplitude. Continuous line is the best fit using the Hill equation (n = 10). The effect of mibefradil (10 μM, holding potential of −100 mV and depolarization to −30 mV, trace marked “M”) is shown on the inset. Rel cur: currents normalized to the current amplitude recorded in the absence of mibefradil.

Low-voltage activated (LVA) Ba^2+^ currents with fast activation/inactivation kinetics could be recorded 2–3 days after injection of Am*-Ca*_*V*_*3* mRNA into Xenopus oocytes, (Fig. 1a). When series of depolarizations were applied to these oocytes, the Ba^2+^ currents started to activate for hyperpolarized potentials close to −70 mV (Fig. 1b), peaked close to −30 mV and reversed at +28.5 ± 3.7 mV (n = 15), with characteristics typical for LVA channels (fast activation and inactivation kinetics and low single channel conductance, see Supplementary Fig. S1a-1). The channels activated rapidly (time to peak of 11 ± 2 ms, n = 40), and had fast kinetics of inactivation with a time constant varying from 150 ms to 15–20 ms between −60 and 0 mV (with an e-fold change per 7.9 ± 0.4 mV, n = 10, Fig. 1c). The steady-state inactivation was complete at potential close to −50 mV (Fig. 1b), and when superimposed with the activation curve, revealed the typical window current found for other LVA channels (Fig. 1b). Recovery of *Am*-Ca_V_3 channels inactivated after a first depolarization at −30 mV was completed after ~1.5 s, following a bi-exponential time-course with fast and slow time-constants of 32 ± 4 and 408 ± 51 ms (n = 10, Supplementary Fig. S1a-2). As expected for LVA Ca^2+^ channels, ionic currents resulting from *Am-*Ca_V_3 expression were not modified by co-expression of the auxiliary HVA Ca^2+^ channel subunit *Am*-Ca_V_β^20^ as current amplitudes (Fig. 1d), current kinetics and current-voltage curves were similar. Finally, when replacing Ba^2+^ ions in the external medium by Ca^2+^ ions (both at 10 mM) the inactivation kinetics were similar in both cases, suggesting a lack of Ca^2+^ -dependent inactivation (see current traces in Supplementary Fig. S1b). Current amplitudes were different, however, with a relative Ca^2+^ to Ba^2+^ and Na^+^ to Ba^2+^ current ratios of 0.63 ± 0.01 and 0.46 ± 0.05 (n = 8 and 5, respectively, Supplementary Fig. S1b). The single channel conductance of *Am-*Ca_V_3 measured in cell-attached mode was low, with a slope conductance of 3.3 ± 0.9 pS in 100 mM Ba^2+^ (n = 4, Supplementary Fig. S1c and d).

Since the pharmacology of the insect Ca_V_3 channels has never been studied before, we decided to test the sensitivity of the *Am-*Ca_V_3 channel to various ion channel agonists/antagonists, classical insecticides (pyrethroids, neonicotinoids, GABA modulators) or known Ca^2+^ channel modulators. Increasing doses of Ni^2+^ applied in the external recording solution rapidly and reversibly inhibited the Ba^2+^ currents flowing through *Am-*Ca_V_3 channels (Fig. 1e and Supplementary Fig. S2a). This block was complete for doses greater than 5 mM, with an EC_50_ of 0.1 ± 0.1 mM (n = 7). Similar results were found for Cd^2+^ with an EC_50_ of 0.2 ± 0.1 mM (n = 8, Fig. 1e). Insecticides targeting sodium channels (Pe: permethrin, Al: allethrin), GABA receptors (Iv: ivermectin, Pi: picrotoxin, Fi: fipronil, 10 μM), nicotinic receptors (Cl: clothianidin, 10 μM) or ryanodine receptors (Ch: Chlorantraniliprol, 10 μM) were all without effect. In contrast, mibefradil (Mi), a known T-type Ca^2+^ channel blocker was effective at 10 μM (with an inhibition of ~80%). The mibefradil analogue NNC55-0398 (NN) also had a similar potency. However, other LVA or HVA antagonists like TTA-A2 (TT, LVA blocker), nifedipine (Ni, L-type Ca^2+^ channel blocker), Bay-K8644 (BK, L-type Ca channel agonist), verapamil (Ve, L-type Ca^2+^ channel blocker), diltiazem (Di, L-type Ca^2+^ channel blocker), amiloride (Am, Na/Ca^2+^ channel blocker) as well as the insect-specific toxin ω-atrachotoxin (At, a HVA Ca^2+^ channel blocker), the ωAga-IV-A (Ag, a P/Q-type Ca^2+^ channel blocker) or SNX482 (SN, a R-type Ca_V_2.3 Ca^2+^ channel blocker) toxins were all ineffective at 0.001–1 mM (Fig. 1e, see methods for doses). The complete dose-response curve for mibefradil indicated an EC_50_ of 3.5 μM (n = 10, Fig. 1f). The effects of mibefradil and NNC55-0398 were completely reversible at the maximum effective dose (20 μM, Supplementary Fig. S2b). At these doses, mibefradil had no effect on honeybee Na_V_1^21^ or on the Ca^2+^ -permeable DSC1-homologous channel (see Supplementary Fig. S2c). Mibefradil, the only drug able to modulate *Am-*Ca_V_3, thus constituted an interesting pharmacological tool to investigate the expression and role of this channel in honeybee tissues.

### Mibefradil toxicity for honeybees

**Figure 2:**
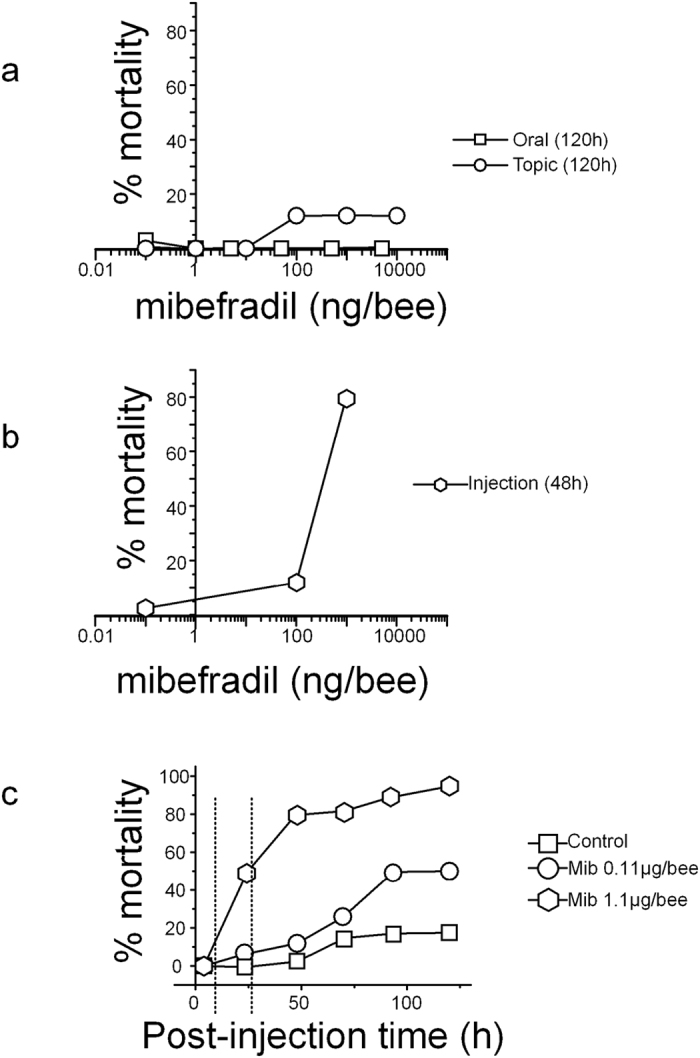
Honeybee toxicity of mibefradil. (**a**,**b**) Dose-mortality curves produced by 3 modes of exposure: oral, topic (**a**) or injection (**b**). Toxicity was evaluated at 120 h for oral or contact exposure or at 48 h post-injection. (**c**) Time course of bee mortality after mibefradil injection at two different concentrations. Mibefradil was injected into the median ocellus at 0.11 μg (circle) and 1.1 μg (hexagon) per bee and the effects were compared to solvent-injected bees (control, square).

Survival to mibefradil was then assessed in three replicates of 13–15 bees at each mibefradil dose. While oral or topic exposures at doses up to 10 μg/bee were almost ineffective (even at 120 h, Fisher exact test, p = 0.612, Fig. 2a), ocellar injection of mibefradil produced a significant mortality after 48 h: from 11.4% at 0.11 μg/bee to more than 70% at 1.1 μg/bee (Fig. 2b). Survival of bees injected with these two mibefradil doses was followed for 120 h (Fig. 2c). At 0.11 μg/bee, there was no (at time <6 h), or low (at 48 h) mortality compared to bees injected with Tyrode (11.4 ± 5.9% at 48 h, Fisher exact test, p = 0.20). At 72 h, mortality was still similar for control and mibefradil-injected bees but started to be significant at 96 h and 120 h reaching 48.9 ± 13.2% (Fisher exact test p < 0.001). At 1.1 μg/bee, mibefradil produced a significant (24 h) to high mortality (79.2 ± 3.3% at 48 h and 94.9 ± 5.1% at 120 h: Fig. 2c). These two doses of mibefradil were thus further used at time 6 h and 28 h post-injection, *i*.*e*. at a time where only sublethal effects are observed at 0.11 μg/bee or moderate mortality for 1.1 μg/bee. In this latter case, the possible behavioral effects on olfactory learning and locomotion were thus analyzed on the surviving bees. It should be noted that control bees, injected with the vehicle only, survived well to harnessing, ocellus removal and injection for two days with mortality not exceeding 2.6 ± 2.6% at 48 h (Fisher exact test, p = 0.33, not significant). Some mortality was only observed the next days (17.9 ± 9.2% at 120 h, Fisher exact test, p = 0.003, significant).

### Mibefradil and olfactory learning

**Figure 3:**
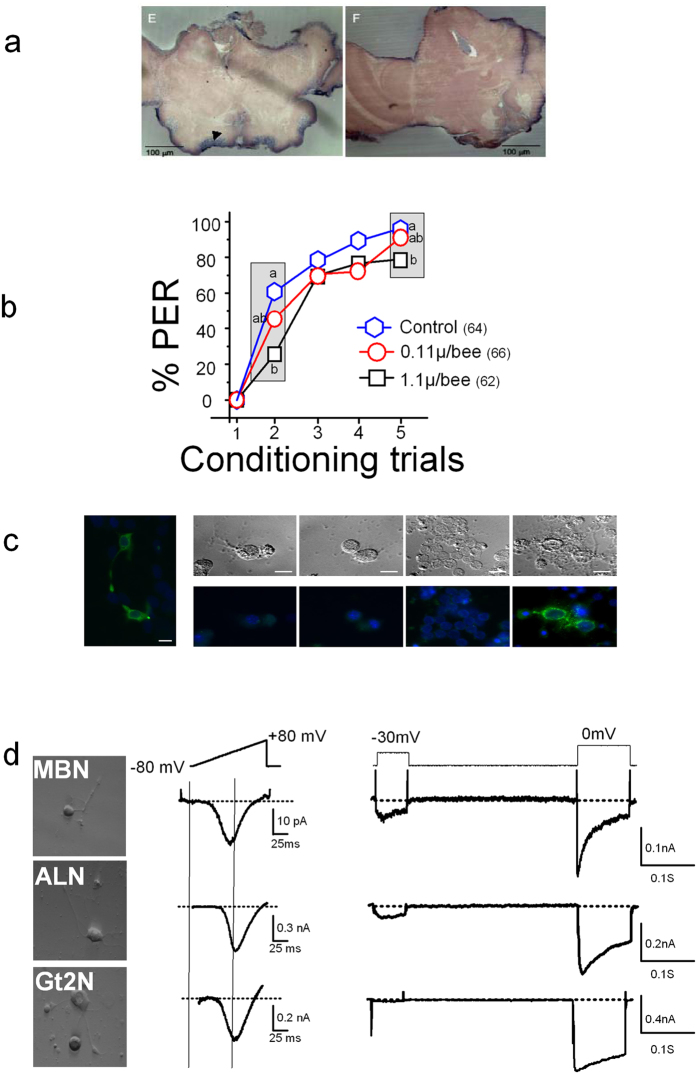
Neuronal Ca_V_3 Ca2^+^ channel and odor learning. (**a**) *In situ* hybridization of frontal section of honeybee brain using an *Am*-Ca_V_3 specific RNA probe. Left, *Am*-Ca_V_3 probe; right, control with the sens probe. The MBN stained by the probe is marked by an arrowhead. Scale bar 100 μm. (**b**) Standard PER conditioning assays performed on bees injected with 1.1 μg/bee mibefradil (black squares) or solvent (blue hexagons) depicted mild deficits in acquisition. At 0.11 μg/bee, mibefradil (red circles) had no effect. See Fig. S4a for tests on 1 h memory and olfactory generalization. (**c**) Staining using Am-Ca_V_3 3CT6 antibody (at 1/100) of Hek-293 cells transfected with the Am-Ca_V_3 or of various honeybee neurons in culture. Staining was performed 2 days after transfection, or at Div 2–6 for cultured neurons. LEFT. Staining of Am-Ca_V_3-expressing HEK 293 cells display a cytoplasmatic and membrane localisation of the channel. RIGHT. Phase contrast images (top) and fluorescent images (bottom) of (from left to right) antennal lobe neurons (ALN), suboesophageal neurons (SBON), mushroom body neurons (MBN) and brain cells other than SBON, ALN and MBN. A few cells amongst the latter brain cells are stained by 3CT6 antibody. Scale bar: 10 μm. (**d**) Ba^2+^ currents recorded on MBN, ALN or Gt2N, during voltage ramps from −80 to +80 mV (middle) or during a two voltage-steps protocol from −100 mV to −30 mV and 0 mV (right). DIC (differential interference contrast) images of neurons where Ba^2+^ currents displayed on the left have been recorded. Scale bar, 10 μm.

An Am-*Ca*_*V*_*3* specific band (MW 685 bp, Supplementary Fig. S3a) was amplified by Rt-PCR on mRNAs isolated from antenna, leg, gut or brain suggesting an ubiquitous expression (see also ref. 12). However, the use of mRNAs from two dissected brain regions (antennal lobes and mushroom bodies), demonstrated that while *Am-*Ca_V_1 and *Am-*Ca_V_2 were clearly expressed in both these regions, the *Am-*Ca_V_3 channel seemed to be more preferentially expressed in the mushroom bodies, although no unambiguous quantification can be obtained from such PCR results (Supplementary Fig. S3a). *In situ* hybridization with specific *Am-*Ca_V_3 RNA probes on brain frontal sections confirmed this suspected regional expression, with a clear signal in the calices of the mushroom bodies (arrowhead, Fig. 3a) where the somata and dendrites of Kenyon cells are located, and almost no signal in other brain areas, suggesting that these regions did not express the LVA Ca^2+^ channels at significant levels.

Olfactory learning performances were then directly tested with a protocol of 5 trials (as described in Supplementary Information) 2.5 h after intra-ocellar injection of mibefradil at 0.11 or 1.1 μg/bee, thus at a time when no lethality was observed. Adult (13 days old) bees from all three groups (vehicle-injected, 0.11 and 1.1 μg mibefradil/bee) did learn to associate the conditioned stimulus odor (CS) with the unconditioned stimulus (US) sucrose, as their proboscis extension response (PER) to the CS increased in the course of training, from 0% (at trial 1) to 77–95% (at trial 5, Cochran’s Q test, Q > 133.0, P < 0.001, 4 df, in all groups). However, acquisition performances were different among groups (Kruskal-Wallis test: H = 11.7, p < 0.01), being lower in the 1.1 μ g mibefradil/bee group than in the control group (Dunn posthoc test, p < 0.01). This difference was significant at trial 2 and trial 5 (Fisher’s exact test, p < 0.001). Note that the reflex (PER) response to sucrose was normal in all three groups of bees (see materials and methods). We conclude that acquisition speed (trial 2) and learning success (trial 5) were slightly affected only by the higher dose of mibefrafil (1.1 μg/bee).

In the generalization tests, performed 1 h after the end of conditioning, bees from all three groups showed a clear generalization gradient, responding differently to the three presented odorants (Supplementary Fig. S4a, Cochran’s Q test, Q > 46.7, p < 0.001, 2 df, in all groups). In the control group, bees responded significantly less to both octanal and 1-hexanol than to the learned odorant, 1-nonanol (Mc Nemar test, p < 0.01), showing that these bees clearly differentiated between the CS and novel odorants. In both groups injected with mibefradil, however, bees responded similarly to 1-nonanol and octanal (Mc Nemar test, p = 0.095 in both cases), and only responded significantly less to 1-hexanol (Mc Nemar test, p < 0.001). This effect could be indicative of mild odor processing difficulties in treated bees compared to control bees, although the olfactory memory *stricto sensu* (i.e. responses to the CS 1 h after learning) was not impaired by mibefradil.

### Am-Ca_V_3 protein is not found in sampled brain and ganglion neurons

A specific anti-Am-Ca_V_3 antibody was prepared and validated on Am-Ca_V_3-transfected HEK 293 cells. A marked staining of perinuclear and membrane regions was obtained, as expected (Fig. 3c-LEFT), while no signal could be obtained on non-transfected cells. When tested against cultured neurons from some brain neuropils involved in olfactory learning and memory (antennal lobes neurons – ALN; mushroom bodies neurons – MBN) or other central neurons (second thoracic ganglion neurons – Gt2N) this antibody yielded no clear staining of these cells suggesting that they did not express a LVA Ca_V_3 channel protein (Fig. 3c). Some cytoplasmic/perinuclear staining was only observed on unidentified neurons resulting from cultures of brain neurons left after dissection of the above neuropils. It thus appeared that, although the Am-*Ca*_*V*_*3* mRNA was produced in MBN, neither a strong effect of mibefradil on olfactory learning nor expression of *Am-*Ca_V_3 at the protein level in ALN or MBN could be unambiguously detected.

**Figure 4:**
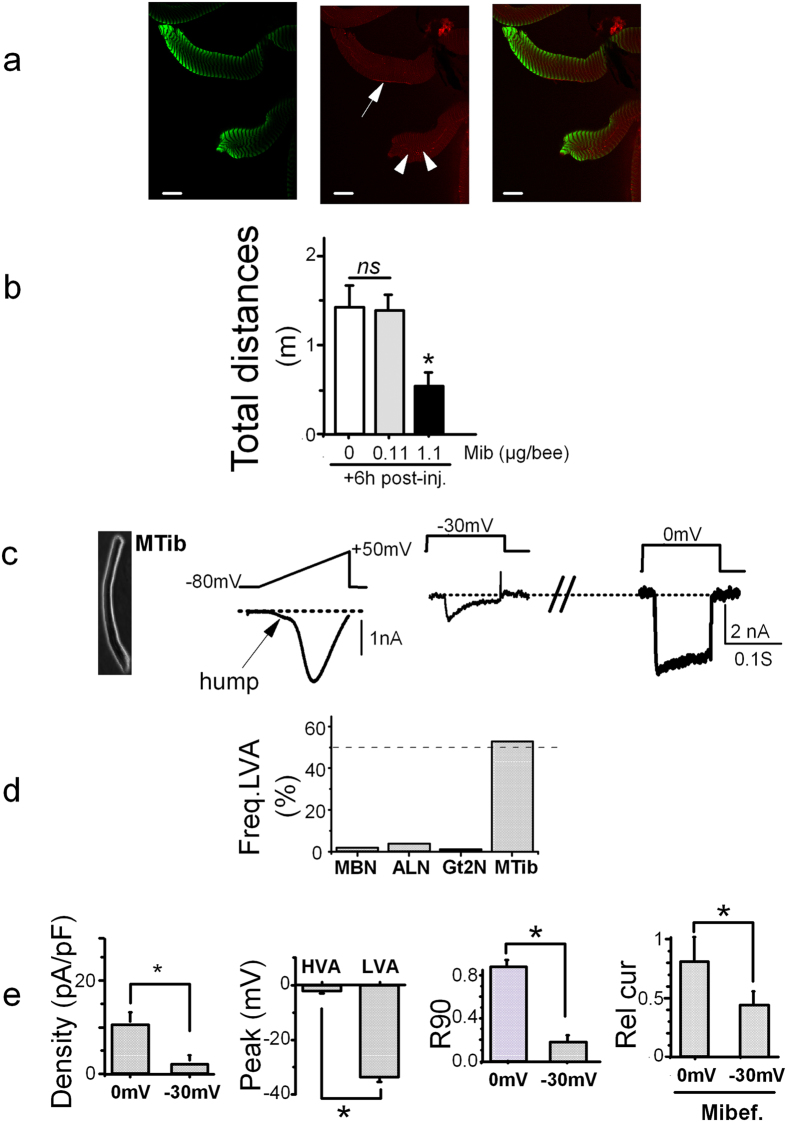
Muscle Ca_V_3 Ca2^+^ channel and locomotion. (**a**) Confocal images of isolated muscle cells stained with phalloïdin (left, green) and with Am-Ca_V_3 specific antibody 3CT6, (middle, red). Merged staining is shown on the right. Ca_V_3 staining is observed in bands within and between phalloïdin staining, in lines along the plasma membrane (arrow) and in isolated spots (arrowheads). The averaged distances ratio of phalloïdin staining over Ca_V_3 staining was 1.6 ± 0.2 (n = 14). Scale bar, 30 μm. (**b**) Averaged walking distances covered by bees during 3 minutes in a vertical circular arena after intra-ocellar injection of 0, 0.11 or 1.1 μg/bee of mibefradil. Experiments were performed 6 h after injection (+6 h post-inj.). (**c**) Ba^2+^ currents recorded on isolated leg muscle (MTib), during voltage ramps from −80 to +80 mV (middle) or during a two-step voltage-clamp protocol with depolarizations from −100 mV to −30 mV and to 0 mV (right). Left: typical DIC (differential interference contrast) images of the muscle cell where Ba^2+^ currents have been recorded. The arrow indicates the presence of a hump (see text) in the IV curve at hyperpolarized potentials. (**d**) Proportion of cells displaying traces with LVA currents evaluated by the presence of a hump on the current-voltage curve for MBN (n = 30), ALN (n = 19), Gt2N (n = 15) and muscle cells (MTib, n = 58) (**e**) Left. Current density of the Ba^2+^ currents recorded in muscle cells at −30 mV or 0 mV ([Ba^2+^] = 2 mM, n = 32). Middle-right. Voltage for peak amplitude of the current-voltage curves for LVA and HVA muscle cell Ba^2+^ currents (n = 16 and 32). Kinetics of inactivation (Middle-left), quantified as the remaining current at 90 ms after the onset of the depolarization (R90), for Ba^2+^ currents recorded in muscle at −30 mV or 0 mV (n = 17). Right. Effect of mibefradil (10 μM) on Ba^2+^ currents recorded on muscle cells (depolarization to −30 mV or 0 mV). Rel cur: currents normalized to the current in absence of mibefradil.

To address this issue, Ba^2+^ currents were recorded on isolated ALN, Gt2N and MBN cultured neurons. As immunostaining suggested a low (if any) *Am*-Ca_V_3 expression in these cells, these recordings were performed in 20 mM Ba^2+^ to increase the signal-to-noise ratio. Voltage ramps (from −80 to +80 mV) or two-pulse recordings at −30 and 0 mV were applied to detect any LVA or HVA channel (see Fig. 3d). The criteria used to assess Ba^2+^ currents through LVA channels were: (i) fast inactivation with activation at a hyperpolarized potential, or (ii) the presence of a hump in the hyperpolarized part of the current-voltage curve, similar to that seen in muscle cells (see ramp in MTib, Fig. 4c).

None of the ALN, MBN and Gt2N neurons tested (n = 15, 25 and 15) displayed such a hump in the current-voltage curves (Fig. 3d) suggesting that these cells did not express any T-type LVA channels. While they did not show any hump on the current-voltage curve, MBN neurons, however, presented a negative shift (toward hyperpolarized voltages) of this curve (Fig. 3d) and current with fast kinetics of activation and inactivation at 0 mV (Supplementary Fig. S3a). Nevertheless, the peak of the MBN current-voltage curve was shifted to less negative voltages (peak at −3.1 ± 1.6 mV) than that of a typical LVA channel and inactivation kinetics, quantified as R90 (0.35 ± 0.03, n = 29), were slower (Fig. 3d and Supplementary Fig. S3) than those of *Am-*Ca_V_3 expressed in a heterologous context (R90 = 0.07 ± 0.01, n = 37, see also Fig. 1a). Moreover, neither Ba^2+^ currents nor KCl-induced variations of intracellular calcium concentration in MBN were sensitive to mibefradil (80 ± 20% of the control Ba^2+^ current, n = 4, see also Supplementary Fig. S3f). Altogether, these data suggested that, while the Ba^2+^ currents recorded in MBN were actually hyperpolarized and rapidly inactivating, the underlying Ca^2+^ channel was not encoded by the *Am-Ca*_*V*_*3* gene.

The neuronal population expressing the *Am*-Ca_V_3 protein could not be recognized in an unstained dish of living neurons, preventing a characterization of functional LVA current in these neurons.

### *Am*-Ca_V_3 is expressed in muscle cells and mibefradil affects locomotion

RT-PCR experiments performed using *Am-Ca*_*V*_*3* specific primers (Supplementary Fig. S3a) and RNA from bee legs revealed expression of *Am-Ca*_*V*_*3* in this tissue. Immuno-histofluorescence analysis of isolated leg (hind leg) muscle using the anti-*Am*-Ca_V_3 specific antibody revealed a patterned staining with *Am*-Ca_V_3 immunoreactive bands in the middle and between the phalloidin staining (Fig. 4a, and Supplementary Fig. S6). Moreover, peri-cellular isolated strips and punctuated intracellular *Am*-Ca_V_3 staining were also observed in some muscle cells (Fig. 4a), while in other cells no clear immunoreactivity could be detected (not shown).

Young bees injected with the vehicle only did not show any evidence of abnormal behaviour (in three replicates of 15 bees), but 1 h after ocellar injection of 0.11 or 1.1 μg/bee of mibefradil, locomotor deficits started to be observed. A simple behavioral observation showed that 20% (9/45) of bees injected with 0.11 μg mibefradil and 90% (39/42) of bees injected with the highest dose (1.1 μg) tended to spontaneously fall and remain upside down, apparently not attempting to perform any of the usual righting reflexes, but none of them died at 6 hours after injection. These deficits were then quantified 6 hours after injection by video-tracking during 3 minutes using a vertical locomotion arena (modified from^22^). The distance covered by control bees (1.45 ± 0.24 m n = 15, Fig. 4b) and bees injected with 0.11 μg mibefradil (1.41 ± 0.18 m, n = 17) was not significantly different, but the fraction of bees remaining in an immobile position were higher with 0.11 μg mibefradil injected bees (6% versus 17%, Supplementary Fig. S4b). When 1.1 μg mibefradil was injected, a major locomotion deficit was observed with a 62% decrease in the covered distance (0.55 ± 0.18 m, n = 15, Fig. 4b) and an increased fraction of immobile bees (80%, Supplementary Fig. S4b). Remarkably, on 13 day-old adult bees, mibefradil injection produced no evident sign of muscular disturbance at 6–10 h post injection, suggesting (1) that the mild perturbation of the PER shown above was not due to muscular defects of the M17 muscle that drive the proboscis (as also suggested by the unaltered proboscis extension reflex to sucrose) and (2) that the locomotion defects are developmentally regulated.

Ba^2+^ current recordings in 53% (n = 38) of the freshly-isolated muscle cells display a typical hump at hyperpolarized potentials when current-voltage curves were constructed using ramp potentials (Fig. 4c and d, Figs S3 and S5) or voltage steps. This hump, typical of LVA channels, was of variable amplitude when compared to the HVA amplitude with an average ratio of 0.33 ± 0.10 n = 8, see Fig. 4e). In these isolated muscle cells, the current inactivation of the LVA channel (at −30 mV) was clearly faster than the one of the HVA current (current traces on Fig. 4c and R90 in Fig. 4e). The current-voltage curves of the LVA Ba^2+^ current (Supplementary Fig. S5b) revealed hyperpolarized voltages for activation (Fig. 4c and e) and reversal potential (26 ± 3 mV, n = 11), when compared with the HVA currents recorded on muscle cells and MBN (39 ± 3 mV, n = 13 for muscle HVA and 45 ± 3 mV for MBN, n = 45, Supplementary Fig. S3c) suggesting slightly different ionic selectivities. The Ca^2+^/Ba^2+^ permeability ratio of LVA Ca^2+^ channels was clearly smaller than for HVA channels in muscle cells (Supplementary Fig. S3d). This Ca^2+^ permeability did not produce any visible sign of Ca^2+^ -dependent inactivation for both types of currents, and the R90 calculated in Ba^2+^ or in Ca^2+^ were quite similar (Supplementary Fig. S3c). Finally, current inhibition with mibefradil (at 10 μM) was more easily seen on LVA currents (more than 50% block) than on HVA currents (Fig. 4e). The HVA current in these muscle cells displayed a typical depolarized current-voltage curve (Supplementary Fig. S5c) that was not further analyzed. Therefore, although it could not be recorded in the somata of motoneurons from the thoracic ganglia, a LVA current was functionally expressed at least in some leg muscles cells.

## Discussion

While HVA Ca^2^ currents have been analyzed in various central and peripheral insect neurons, in adults and larvae of *Drosophila*, honeybee and cockroach^2^,^4^,^17^,^23^,^24^,^25^,^26^,^27^,^28^, unambiguous LVA T-type Ca^2+^ channels have rarely been recorded. In fact putative LVA currents were recorded in two preparations only: *Drosophila* antennal lobe projection neurons^3^ and *Drosophila* or cockroach motoneurons^15^,^16^. In all these cases, the pharmacological susceptibility of these insect LVA channels, like that of HVA channels, appeared to be rather low, with typical blockers being only effective at hundreds of micromolars. Similarly, while the *Ca*_*V*_*3* gene has been identified in various insects, its heterologous expression has just been reported^14^, but its potential role remained unexplored. This work therefore shows for the first time both heterologous expression of the *Apis mellifera* Ca_V_3 channels, and the characterization of these LVA T-type Ca^2+^ channels in honeybee muscle and their absence in various neuronal preparations.

The recorded biophysical parameters clearly identified a LVA T-type Ca^2+^ channel similar to the *Dm-*Ca_V_3^14^ or *Hs-*Ca_V_3.2^29^ Ca^2+^ channels, with a low Ca^2+^/Ba^2+^ permeability. Vertebrate LVA Ca^2+^ channels are known to be insensitive to dihydropyridines and agatoxins, but strongly blocked by amiloride, mibefradil or TTA2^14^. In honeybee only mibefradil inhibited Ca_V_3 channels with efficacy, while being ineffective on *Am*-Na_V_1 and *Am*-Ca_V_4^30^(bee orthologues of the *para* and DSC1 channels, respectively). Mibefradil is therefore an interesting tool to challenge the role of Ca_V_3 in the honeybee physiology and behavior.

Our data suggest that *Am-Ca*_*V*_*3* is transcribed in various tissues but is only translated in muscle cells and some isolated but unidentified neurons. Expression of Ca_V_3 channels in freshly isolated muscle cells had never been reported before and is confirmed here by electrophysiology. These channels were found to be localized in large bands at the border of the I band, close to the T-tubule^7^,^19^, but also at the cell membrane in long strips of several μm. The identification of these latter structures in adult muscle is under way, but we propose that they may correspond to presynaptic terminals, suggesting that Ca_V_3 could also be expressed presynaptically in motoneurons. This has already been suggested in *Drosophila*^15^ as well as the potential role of Ca_V_3 channels in synaptic transmission in vertebrates^31^. The T-type LVA channel activity was only seen in ~50% of the muscle cells, while HVA channel activity was present in almost 100% of them (see also refs 7 and 19). We do not know at present whether this LVA channel expression has a particular function (fast or slow-contraction, for example) or if it occurs at different developmental stages. Such developmentally-regulated transcription has never been investigated in insects, but is clearly documented in mammalian heart and muscles for T-type channels. When expressed, the Ca_V_3 channels could participate in the early phase of the depolarization, instead of the fast Na^+^ channels that are not present in insect muscles^7^,^19^ and thus tune the cellular excitability. Further work will attempt to decipher more precisely the role of Ca_V_3 channels at different developmental stages.

Patch-clamp recordings in motoneurons from thoracic ganglia did not allow to record any LVA current, although Ca_V_3 activity has been clearly described in *Drosophila* and cockroach motoneurons *in situ*^15^,^16^. This suggests that either (1) *Am-*Ca_V_3 was not expressed in bee motoneurons, or (2) *Am*-Ca_V_3 was expressed at the presynaptic terminals only, as suggested by our immunostaining of muscle and thus channel activity was lost when recording in neuronal culture at DIV2-6 or (3) *Am-*Ca_V_3 expression was developmentally regulated, occurring at the early stages of differentiation, and disappearing in later stages. However, the expression of *Am-*Ca_V_3 channels shown here in muscle cells can explain at least in part the locomotion deficit observed on young, mibefradil-injected, bees.

The lack of *Am-*Ca_V_3 channel expression in ALN and MBN is more puzzling. Current recording and immunostaining on cultured neurons did not allow the identification of any LVA channels in these cells types, but *in situ* hybridization clearly localized messenger RNAs to the calices of the mushroom bodies. In *Drosophila*, Ca_V_3 expression in the brain is wide, although seemingly restricted to the dendrites, and invalidation of the Ca_V_3 channel produces a very mild phenotype, with slightly perturbed vigilance state^14^. In our case, the fact that cultured neurons, at DIV 5–8, had very few elongated dendrites may explain the lack of LVA current in our recordings and the lack of visible *Am-*Ca_V_3 staining. Although we have not attempted to separate interneurons from projection neurons in our ALN cultures, no previous studies identified LVA channels in recordings performed on honeybee or other insect MBNs or ALNs, or even on olfactory neurons, either in culture or *in situ*^2^,^4^,^5^,^6^. This suggests that Ca_V_3 channels may play a minor neuronal role in insects. As expected, injection of high doses of mibefradil in *Apis mellifera* brain, only slightly affected olfactory learning, memory and generalization. Some of the effects we observed could be due to an inhibition of Ca_V_3 channels in the other neuron types that showed the Ca_V_3 positive staining described here. Unfortunately, despite several attempts, we could not obtain any Ca_V_3 recordings from these neurons in culture. In addition, the fact that mibefradil could partially inhibit HVA channels in muscle, but hardly affected HVA in MBNs, suggests that Ca_V_2 and Ca_V_1 are differentially affected by the drug and that part of the effect of mibefradil in neurons could be due to Ca_V_1 inhibition. Clearly, the characterization of the effect of mibefradil on Ca_V_1 and Ca_V_2 channels expressed in Xenopus oocytes represents a next step to fully understand its effects *in vivo*. In any case, the role played by *Am-*Ca_V_3 in neuronal functions, either at somatic or presynaptic location, appears to be moderate, at least at the developmental stage where learning has been tested (*i*.*e*. 13 day-old forager bees).

In conclusion this work demonstrates the functional expression of Ca_V_3 LVA channels in honeybee muscle cells, and its potential role in muscular function. Ongoing studies are now focused on the role of this channel during muscle development. These results also suggest that the role played by *Am*-Ca_V_3 channels in the nervous system appears to be quite modest.

## Methods

This study was carried out in strict accordance with the recommendations and relevant guidelines of our institution. Surgery was performed under anesthesia, and efforts were made to minimize suffering. The care and use of Xenopus conformed to institutional policies and guidelines. The experimental protocols were approved by the Direction Départementale des Services Vétérinaires (authorization N° C34.16).

### Antibodies

Rabbit polyclonal antibodies were generated against synthetic peptides corresponding to the specific *Am*-Cav3 N-terminus (NH2-CFDVPSSDGPSDPS-CONH2). Sera were collected and antibodies were affinity purified against the immunization peptide. Their specificity was checked by western-blot using cellular extracts from Hek 293 cells transfected with the *Ca*_*V*_*3* gene or from honeybee brain tissues, and by immunofluorescence on transfected cells, cultured neurons or brain slices.

### Cell culture - Electrophysiology -Behavior

Recording Ca_V_3 Ca^2+^ channel activity in honeybee neurons and Xenopus oocytes, *in situ* hybridization, immunofluorescence, locomotion evaluation and proboscis extension reflex (PER) were done as described in Supplementary Information.

### Solutions for cell culture and electrophysiology

The Ca^2+^ and Mg^2+^ -free Tyrode used for honey bee muscle and neuron dissection contained (in mM): 140 NaCl, 5 KCl, 10 HEPES, 90 sucrose (pH 7.2, adjusted with NaOH, 400 mOsm/L). The hyperosmotic Tyrode contained 190 instead of 90 mM sucrose. Culture medium was made of a commercial liquid L15 medium (with L-glutamine) supplemented with 5.5 mM D-Glucose, 3.3 mM L-proline, 75 mM sucrose, 10% fetal bovine serum, 1% penicillin/streptomycin (pH 7.2, adjusted with HCl, 400 mOsm/L). The standard extracellular solution used for whole-cell recording of neurons was (in mM): TEA-Cl, 140; MgCl_2_, 2; BaCl_2_, 20; HEPES, 10; sucrose, 100 (pH was adjusted to 7.2 with TEAOH and the osmolarity was ~500 mOsm with sucrose). The pipettes had a resistance of 5–10 MΩ when filled with the following solution: CsCl, 135; NaCl, 5; MgCl_2_, 1; EGTA, 10; HEPES, 10; sucrose, 190 (pH adjusted to 7.2 with CsOH, and the osmolarity was ~480 mOsm with sucrose). For the recordings of honeybee muscle cells, the following extracellular solution was used (mM): TEAOH, 120; MgCl_2_, 2; BaCl_2_, 2; 4-AP, 1; HEPES, 10; pH 7.2 (adjusted with MeSO_3_). The pipettes (1–3 MΩ were filled with the following solution: Kgluconate, 140; MgCl_2_, 2; EGTA, 10; HEPES, 10 (pH adjusted to 7.2 with KOH, osmolarity adjusted to 300 mOsm) in order to suppress the sodium current. For two-electrode voltage-clamp recording of Xenopus oocytes, pipettes were filled with 3 M KCl, and the BANT10 recording solution contained (in mM): BaOH, 10; TEAOH, 20; NMDG, 50; CsOH, 2; HEPES, 10; pH 7.2 with methanesulfonic acid (see Supplementary Information for details. For recording of single channel activity of cell-attached patches of Xenopus oocyte membrane (Fig. S2C and D) the pipette was filled with (in mM): BaCl, 100; Hepes, 5; pH adjusted to 7.2 with NaOH, and the depolarizing bath solution was (in mM): KCl, 100; HEPES, 10; pH adjusted to 7.2 with KOH (see Supplementary Information for more details).

Values are given as mean ± S.E.M. Student t-test or ANOVA were used to assess the differences between mean values, with a significance level set at *P* < 0.05.

## Additional Information

**How to cite this article**: Rousset, M. *et al*. Honeybee locomotion is impaired by Am-Ca_V_3 low voltage-activated Ca^2+^ channel antagonist. *Sci. Rep.* **7**, 41782; doi: 10.1038/srep41782 (2017).

**Publisher's note:** Springer Nature remains neutral with regard to jurisdictional claims in published maps and institutional affiliations.

## Supplementary Material

Supplementary Information
