# Supplementary material for: Honeybee locomotion is impaired by Am-CaV3 low voltage-activated Ca2+ channel antagonist
Source: Sci Rep. 2017 Feb 1;7:41782. doi: 10.1038/srep41782 (PMC5286435; doi:10.1038/srep41782)
Supplement: Supplementary Information [file srep41782-s1.pdf]

Supplementary information

Honeybee locomotion is impaired by Am-Ca_V3 low voltage-activated Ca^{2+} channel antagonist

Rousset M[#], Collet C[#], Cens T[#], Bastin F, Raymond-Delpech V, Massou I, Menard C, Thibaud J-B, Charreton M, Vignes M, Chahine M, Sandoz J-C, Charnet P
[#]: equal participation to this work

SUPPLEMENTARY METHODS

This study was carried out in strict accordance with the recommendations and relevant guidelines of our institution. Surgery was performed under anesthesia, and efforts were made to minimize suffering. The care and use of *Xenopus* conformed to institutional policies and guidelines. The experimental protocols were approved by the "Direction Départementale des Services Vétérinaires" (authorization N° C34.16).

Xenopus oocytes and two-electrode voltage-clamp

Xenopus oocytes were prepared and injected with *in vitro* transcribed RNA (20-40 nl of *Am-*Ca_V3 (Genbank acc. numb KJ485706) +/- *Am-*$Ca_V\beta a$ (JX997991) at 1 µg/µl, (stoichiometry 1:1) as already described (1).
Macroscopic currents were recorded under two-electrode voltage-clamp and analyzed in the BANT10 recording solution (in mM): BaOH (10), TEAOH (20), NMDG (50), CsOH (2), HEPES (10), pH 7.2 (adjusted with methanesulfonic acid). Current-voltage curves were obtained either from staircase protocols (150 ms-long pulses from -120 mV to 50 mV, with 10 mV increment) or using voltage ramps ranging from -100 mV to +40 (1.3 V/s).
Currents were filtered (500 Hz) and digitized (2 kHz) using a Digidata-1200 interface (Axon Instruments). Data acquisition was done using version 7 of the pClamp software (Axon Instruments). Around 30 nl of a BAPTA solution (in mM: BAPTA free acid (100), CsOH (10), HEPES (10), pH 7.2 adjusted with CsOH) were micro-injected into each oocyte with an home-made pneumatic device (pulse of a 10 psi positive pressure for 150 msec) at the beginning of the recording, using a third electrode, to minimize the Ca^{2+}-activated Cl^- current.
Current to voltage curves normalized to the peak current Imax were fitted (least squared fitting) using the following equation:

$$I/Imax= G*(V-E_{rev})/(1+exp((V-V_{act})/k_{act}))$$

where I is the current amplitude, Imax is the peak current amplitude measured at the maximum of the current-voltage curve, V is the value of the applied membrane potential , and where E_{rev} (the apparent reversal potential), G (the normalized macroscopic conductance), V_{act} (the potential for half-activation), and k_{act} (a slope factor) are 4 adjustable parameters.
Isochronal steady-state inactivation curves (2.5 s of conditioning voltage followed by a 400 ms test pulse to -30 mV) were fitted using the following equation:

$$I/Imax=R+(1-R)/(1+exp((V-V_{in})/k_{in}))$$

where I is the current amplitude measured during the test pulse to -30 mV for conditioning voltage steps varying from -80 to +50 mV, Imax is the current amplitude measured during the test-pulse for a conditioning step to -80 mV, V is the conditioning voltage and R (the proportion of non-inactivating current), V_{in} (the potential for half-inactivation) and k_{in}(a slope factor) are the three adjustable parameters.

Inactivation kinetics were quantified as R90, which is the ratio of the peak current amplitude over the current recorded at the end of a 90 ms depolarization. Current traces were also fitted using a mono-exponential equation giving the value of the time-constant of inactivation (noted Tau, Figure 1c). Kinetics of channel reactivation was challenged with two depolarizing pulses at -40 mV separated by a resting period at -120 mV of a variable duration. The channel recovery time course was estimated from the % of current recovery at the 2nd pulse expressed as a function of the duration of the resting interval (figure S2A-2 for current traces). This recovery curve was then fitted with a biexponential function giving the characteristic fast and slow time constants for channel recovery from inactivation.

***Single channel recordings*.**

For single-channel recordings (figure S2 C and D), the oocyte vitelline membrane was removed using forceps after immersion in a hypertonic solution (200 mM NaCl, 10 mM HEPES, pH 7.2 adjusted with NaOH) and the oocyte was then placed in the recording chamber filled with a depolarizing solution (100 mM KCl, 5 mM HEPES, 10 mM EGTA, pH 7.2 adjusted with KOH; the osmolarity was ~250 mOsm). Coated (Sylgard®) and fire-polished patch-pipettes had a resistance of 8–12 MΩ when filled with the pipette solution containing 100 mM $BaCl_2$, 5 mM HEPES (pH 7.2 was adjusted with NaOH; ~290 mOsm). Cell-attached patch-clamp currents were recorded with an Axopatch 200B amplifier (Molecular Devices), low-pass filtered at 2 kHz and digitized at 10 kHz using a Digidata 1200 interface and stored on a computer using the Clampex software. The liquid junction potential was 1–3 mV and was thus neglected. Currents were analyzed with the Clampfit software (ver 10, Molecular Devices). Linear leak and capacitive transients were subtracted by means of the manual baseline adjustment command of Clampfit. Well-resolved channel openings were detected by a threshold analysis set at 50% of the elementary current. Channel conductance was calculated from Gaussian fits of amplitude histograms, obtained at different voltages. Mean ensemble currents were calculated by averaging ~50 consecutive traces.

Bees

Newly emerged bees (*Apis mellifera*) were obtained during the spring season from single hive maintained in the experimental apiary of the "Abeilles & Environnement" research department, on INRA-PACA campus in Avignon, France. Colonies received a treatment against Varroa in October (Apivar™, active ingredient amitraze) and were healthy, without any obvious symptoms of disease. To collect bees, frames of developing brood were gently brushed to get rid of adult bees and placed into an incubator (30°C, high humidity) overnight in order to harvest newly emerged bees the next morning. Upon emergence, these bees were kept in cages at 29°C , under high humidity and fed with food (candi) stored in combs (see (2)).

Primary cell culture and patch-clamp

Apis mellifera neurons. Mushroom body neurons cells (MBN), antennal lobe neurons (ALNs) and neurons from the second thoracic (Gt2N) ganglia were isolated from honeybee *A. mellifera* pupae (at stages between 4 and 6 days before emergence). Pupae were first dipped in alcohol for a few seconds and then rinsed in sterile distilled water for sterility purposes.

The brain was dissected out of the insect's forehead in a sterile Ca^{2+}- and Mg^{2+}-free Tyrode (400 mOsm/l, see Solutions). The brain sheath was then removed and the antennal lobes, thoracic ganglia and mushroom bodies were isolated. After a hyperosmotic non-enzymatic dissociation in Ca^{2+}- and Mg^{2+}-free Tyrode, (500 mOsm/l, 4°C; 15 min) and centrifugation (0.3 g, 3 min), the pellet was suspended in culture medium (see Solutions). Fragments of the antennal lobes, thoracic ganglia or mushroom bodies were gently triturated through the disposable tip of a 100 µl pipette. Isolated neurons were plated on poly-L-lysine coated plastic Petri dishes, and cultured in an incubator (29°C, high humidity). All experiments were done on 1 to 6 day-old cell (Days in vitro –DIV- 1-6) cultures and performed at room temperature (20-22°C).

Whole–cell currents were recorded in voltage-clamp conditions with an Axopatch 200B amplifier (Molecular Devices), low-pass filtered at 2 kHz and digitized at 10 kHz using a Digidata 1200 interface

(Axon Instruments). Current and voltage were stored on a computer using the Clampex software (ver. 7.03). Currents were recorded in 20 mM external Ba^{2+} (or Ca^{2+}, when noted). Cell capacitance and series resistance were compensated to ~70-80%, and the leak current was subtracted using the P/5 procedure. Current amplitude was measured at the peak of the current recorded during a 90-150 ms-long test potential to -30 mV or 0 mV (from a holding potential of -100 mV), and the kinetics of inactivation were quantified by the ratio of the peak current over the current amplitude recorded at the end of the pulse (R90 or R400 for neurons or muscle cells, respectively).
Current-voltage curves where obtained using voltage ramps from -80 mV to +80 mV (2.1 V/s) applied to honeybee neurons and muscle cells. The recorded current traces with a discernable hump were then fitted with a function representing the summation of 2 current-voltage curves:

$$I= (Imax1*G1)*(V-E_{rev1})/(1+exp((V-V_{act1})/k_{act1})) + (Imax2*G2)*(V-E_{rev2})/(1+exp((V-V_{act2})/k_{act2})),$$

with parameters for the two curves having the same signification as above(*Xenopus* oocyte section), the 8 adjusted parameters being here the two apparent reversal potentials (E_{rev1}, Er_{ev2}), the two (Imax*G) products, V_{act1}, V_{act2}, k_{act1} and k_{act2}. The fitting procedure, performed by limiting the reversal potentials to values greater than 10 mV, provided the typical parameters for the two curves. Each curve was then individually reconstructed, and the peak values (I_{max1} and I_{max2}) were determined for each curve and used for the calculation of the mean LVA and HVA current amplitudes in muscle.

[Ca]$_i$ measurements

Intracellular calcium concentration ($[Ca^{2+}]_i$) was measured with the fluorescent indicator fura-2. For this purpose, mushroom body neurons (mainly Kenyon cells) grown on square (10 × 10 mm) glass coverslips were loaded with fura-2 by a 30-min incubation at 33°C with 1 µM fura-2-AM and 0.04 % Pluronic (Sigma) in the extracellular solution: 124 mM NaCl, 3.5 mM KCl, 25 mM $NaHCO_3$, 1.25 mM NaH_2PO_4, 1 mM $CaCl_2$, 2 mM $MgSO_4$, 10 mM D-Glucose and 10 mM HEPES (pH 7.4). $[Ca^{2+}]_i$ was monitored by videomicroscopy. After rinsing, a glass coverslip was transferred to the recording chamber mounted on an inverted microscope (Leica, DMIRB). Fura-2 emission was obtained by exciting alternatively at 340 and 380 nm with a rotating filter wheel (Sutter Instruments) and by monitoring the resulting emissions (F340 and F380) at 510 nm. The ratio of emissions at 510 nm (F340/F380) was recorded every 2 s. Fluorescent signals were collected with a CCD camera (Hamamatsu), digitized, and analyzed with image analysis software (Acquacosmos, Hamamatsu). The coverslips were continually superfused with the extracellular solution. Drug application (KCl 30 mM +/- mibefradil 10 µM) was performed with a gravity-fed system.
The "n" values represent the entire population of cells recorded in at least 3 independent cultures. Data are presented as means ± S.E.M. on graphs where pooled data are plotted.

RT-PCR

RNA preparation: tissues were homogenized in 500-1000 µl of Trizol (TRIzol® Reagent, Ambion), and incubated 5 min at room temperature (RT). 100-200 µl of chloroform were added, followed by a 5 min incubation at RT, and a 10 min centrifugation at 12000g at RT. 250-500 µl of supernatent were diluted at ½ with isopopanol, incubated 10 min and centrifuge 10 min at 12000g at RT. The pellet was then washed with 500 µl of 75% ethanol, resuspended in desionized water and RNA concentration was evaluated.

Rerverse transcription reaction (2-5 µg of RNA; 2 µl of a 100 µM Oligo-dT(18) solution; 2 µl of 10 mM dNTP; 2 µl of 100 mM DTT; 4 µl of 5x First Strand Buffer (Invitrogen)) was incubated 5 min at 65°C. Then, 1 µl RNase Inhibitor (40 U/µl, Fermantas), 1 µl MMLV-reverse transcriptase (200 U/µl, Invitrogen) were added and incubated 60-90 min at 37°C and 15 min at 75°C. Finaly 1 µl RNaseH (5 U/µl, New England Biolabs) were added and incubated 20 min at 37°C.

The 50 µl PCR reaction contained: 1-2 µl of RT; 1 µl of sens and antisens oligonucleotides at 100 µM; 10 µl 5x Herculase Buffer (Agilent); 1 µl of 10 mM dNTP; 1 µl Herculase II fusion DNA Polymerase (Agilent); volume adjusted to 50 µl with H_2O . The reaction was incubated for 2 min at

92°C, followed by 35 cycles [30 s at 92°C, 30 s at 56°C, 1 min at 72°C], and a final 5 min incubation at 72°C. 2% agarose gels were loaded with 10 µl of each reaction.
Here below are the oligonucleotides designed to amplify in each gene sequences that are not subject to alternative splicing :
amCav1-031S: 5'GATTGGGCAAGTACTGCGATCCG3'
amCav1-012AS: 5'GAACACTAGAATCTATCGGCCGATGG3',
with a size of the amplified fragment of429pb localized in the last exon.
amCav2-003S: 5'GCTGCTCAGCTCGATGCGTAGC3'
amCav2-011AS: 5'CCTCCTCTTCCTCCTCGTTCTCGG3',
with a size of the amplified fragment of 383pb localized at exon 12/13
amCav3-013S: 5'AAGAGAATTGGCCGCAGAGCA3'
amCav3-014AS: '5TGGCCTGCAACTCAACGTTG3',
with a size of the amplified fragment of 685pb localized in the last exon (3).

In situ hybridization

In situ hybridization was performed using digoxygenin (DIG) (Roche, France) labelled RNA probes on whole brains. Probes of 744 nt for Ca_V3 (nt 3257-4001) were amplified by PCR using primers containing T7 or SP6 promoting sequence for respectively antisens or sens probes.
Honeybee heads were isolated and fixed in 4% paraformaldehyde (PFA) in PBS during 1 hour. Bee brains were then dissected, post-fixed in PFA overnight at 4°C. Brains were rinsed in PBTX (PBS in which 0.1% Triton X100 was added) and then dehydrated with varying concentrations of methanol. Brains were pretreated for 100 s at 20°C in proteinase K (1:2000 in PBTX) and postfixed for 20 min on ice in 0.2% glutaraldehyde, 4% PFA. Brains were then incubated at 62°C in prehybe solution overnight. Denaturized probes were applied on the brains (1 µg/ml) and incubated overnight. Following post hybridization washes and blocking, brains were incubated overnight in 1:2500 anti-DIG antibody, 10% Normal Goat Serum, 2% BSA. Washes were conducted at 4°C with PBTX and 0.1% BSA and kept overnight in this solution. Brains were stained 24 h with nitro blue tetrazolium/5-bromo-4-chloro-3-indolyl phosphate (Roche Applied Science) in NTMT buffer (100 mM NaCl, 100 mM Tris-HCl pH 9.5, 50 mM $MgCl_2$).
Honeybee brain inclusions were made in Gelatin Albumin Mixture added to glutaraldehyde. Sections of 30 µm, obtained using a vibratome, were mounted in Mowiol® (Sigma-Aldrich) to allow microscopy observations.

Immunofluorescence

HEK-293T cells were grown and transfected as described (1). 48 h after transfection, cells were fixed with PFA (4% in PBS) and permeabilized (0.25% Triton X-100 in PBS) and processed for immunofuorescence. An antibody raised against Am-Ca_V3 was used to visualize the subunit localization by confocal microscopy (RIO Imaging facility, Montpellier).

Honeybee brains were dissected at 4°C in PBS supplemented with PFA 4% and fixed in the same solution during 2.5 h. After rinsing in PBS, they were successively incubated in 15% and 30% sucrose during 4 h and overnight respectively. Brain slices (25 µm thick) were made and permeabilized with 0.1% Triton-X100 in PBS and blocked with PBS-0.1% Triton X100-0.5% BSA and 10% NGS. They were then incubated for 2 h at room temperature with the Am-Ca_V3 antibody at 1/1000 dilution in PBS-0.1% Triton X100 and 10% NGS. After washing with PBS, the reaction was blocked with PBS-0.5% BSA and 10% NGS. In the case of immunofluorescent revelation secondary antibody, anti-rabbit IgG-TRITC were applied at 1/100 for 1.5 h in PBS-1% BSA. After washing in PBS, slices were mounted in Mowiol and observed under the microscope. In the case of peroxidase–based system, biotinylated anti-rabbit antibody was applied at 1/500 in PBS/10% NGS. The VectaStain ABC kit (Vector Laboratories, USA) was used according to the manufacturer recommendations. After rinsing in PBS the slices were incubated with 3,3'-diaminobenzidine (DAB) solution added with 3% $NiCl_2$. The reaction was blocked with PBS- 0.05% azide sodium and the slices were dehydrated by

incubation in successive ethanol solutions (from 50% to 100%). After rinsing with limonene, the slices were mounted using EUKITT® mounting medium (Kindler, Germany).

Locomotion evaluation

Newborn bees were collected, fed and kept in cages as previously described (2). Bees in the first day of their imaginal life were anaesthetized in glass flasks dipped a few minutes in ice and subsequently harnessed in vertical plastic tubes. Their proboscis was free to move but their neck was secured with tape to prevent head movements. Before surgery, bees were fed with 5 µl of a 30% (w/v) sucrose solution. The dorso-median ocellus was gently removed under a stereomicroscope with a fine entomological pin. Intra-ocellar injection was achieved using a Hamilton syringe (NanoFil 1 µl) mounted on a micromanipulator. Each bee was injected with 200 nl solution. All solutions were prepared in a Tyrode's solution prepared with sterile distilled water and afterward filtered with a 0.2 µm filter. This solution contained (in mM): 140 NaCl, 5 KCl, 2 MgCl2, 10 HEPES (pH 7.2, 300 mOms/l, without Ca^{2+}). Mibefradil was dissolved at 10 mM and 1 mM in Tyrode's solution (*i.e.* 5.68 g/l, corresponding to 1/5 and 1/50 of the theoretical maximal mibefradil solubility in water respectively). Final mibefradil doses injected were thus 0, 0.1 and 1 µg/bee. After injection, bees were returned to cages (food and water provided *ad libitum*) and placed in a ventilated incubator (29°C, 40% humidity, dark). Six hours after injection, locomotor abilities/deficits were explored in a vertical locomotion arena equipped with a set-up of video-tracking (modified from (2)). In brief, bees were transferred into Petri dishes (9 cm x 1.5 cm in diameter and depth respectively) and locomotor activity was monitored for 3 minutes using a webcam controlled with VirtualDub (GNU free software, frame acquisition frequency 1 Hz, http://sourceforge.net/projects/virtualdub/files/). This arena set-up allowed video tracking of 9 bees at a time (3 rows of 3 dishes attached to a vertical 30x30 frame). Dishes were placed in a closed chamber and illuminated from above to avoid any inluence of daylight variation. The light source consisted of two parallel flicker-free LED ramps (length 10 inches, 9 LED each), for a total of 0.72 W, 70 lumens of cold light (StarLED sticks, Starlicht, Germany). Videos were manually analyzed using Image J (open source, Rasband WS, National Institutes of Health, Bethesda, http://imagej.nih.gov/ij/) with an available plugin in order to obtain a series of x,y coordinates for each bee. Individual paths were analyzed with Excel and the total distance covered by individual bees was measured as an index of the locomotion performance.

Proboscis extension reflex (PER) conditioning

Forager bees were caught in the morning, were fed, and then chilled on ice until they stopped moving. They were harnessed individually in metal holders, leaving their antennae and mouthparts free. Harnessed bees were then injected in the median ocellus with 0.1 µl of 2 mM or 20 mM mibefradil (0.1 or 1 µg/bee) solution, or with vehicle (control group). Finally, they were left for 2.5 h in a dark and moist container until conditioning. Bees were trained to associate an odorant (the conditioned stimulus, CS) with sucrose solution (the unconditioned stimulus, US) delivered to the antennae and proboscis, in a standard PER conditioning protocol (Matsumoto et al. 2012). During the *acquisition phase*, bees received 5 conditioning trials with 10 min inter-trial intervals. One hour after conditioning, bees were then subjected to a *generalization phase*, aimed at studying their responses to novel odorants that are increasingly chemically-dissimilar to the CS. In these tests, the odorants were presented to the bees without reward. Bees were thus presented with the CS (1-nonanol) and with 2 novel odorants (octanal and 1-hexanol) in a random order. At the end of training, bees' PER to sucrose applied on the antennae was tested again, to check their motivation. Bees that did not respond to sucrose at more than half of the trials during conditioning or at this last test were excluded from the analysis (5.9%, without any difference among groups).

Dichotomous PER responses were analyzed using non-parametric statistics. To evaluate the variation of responses within group, Cochran's Q test was used. The performances of different groups of bees during the whole acquisition were compared using a Kruskal-Wallis test, followed when significant by Dunn post hoc tests. Comparisons between groups of responses at a given trial were made using Chi^2 tests for 3 groups, or Fisher's exact test for 2 groups.

Supplementary Figure S1. Caracterization of *Am*-Ca_V3 expressed in *Xenopus* oocytes.

a-1. Average normalized current–voltage curve recorded from *Am-Ca_V3* RNA-injected oocytes (n=16). The curve was normalized to the peak current recorded at voltage between -40 and -20 mV. Fitting using the equation mentioned in material and method section yield a voltage for half-activation (V_{act}) of -47±1 mV, a slope of 6.7±0.3 mV, and a reversal potential (E_{rev}) of 28±4 mV. **a-2.**The reactivation protocol was built using a two pulses protocols to -30 mV from -120 mV (100 ms duration) separated by a resting period of increasing duration (from a few ms to several seconds). The reactivation curve (amount of recovery versus interpulse duration) shown here was then fitted using a two exponential time-course giving two time-constants of recovery: 22±7 ms and 411±42 ms (n=10).

b. Left. Comparison of current amplitudes and kinetics with Ba^{2+} or Ca^{2+} as charge carriers. Oocytes were injected with BAPTA to prevent activation of the endogenous Ca^{2+}-activated Cl^- current, and currents were recorded using a recording solution containing either 10 mM Ba^{2+} or 10 mM Ca^{2+} during step depolarisations to -30 mV. The Ca^{2+} current were clearly smaller than the Ba^{2+} current at the peak current and the averaged current ratios ICa/IBa were around 0.61±0.01 (n=8). The kinetics of current inactivation was evaluated by the R90 ratio (see text) and was Ca^{2+}-independent. **Right.** Current-voltage curves obtained from currents recorded during voltage ramps from -100 mV to +40 mV in the presence of 10 mM external Ba^{2+} (Ba) or Ca^{2+} (Ca) or in 100 mM Na^+ (Na).

c. Top. Single-channel current traces of *Am*-Ca_V3 recorded in oocytes in cell-attached mode using a 100 mM Ba^{2+} solution in the patch pipette and applying depolarizations from -100 mV to -30 mV. Bottom: The current trace, obtained by averaging 50 single-channel traces such as those shown on the top, peaked in 10 ms and displayed a relatively fast inactivation.

D. Representative current-voltage curve for *Am*-Ca_V3 Ca^{2+} channels constructed from single-channel traces similar to those shown on part B, but for various depolarizing steps from –50 to -10 mV. The open channel current amplitude recorded during each depolarization potential was calculated from a two-gaussian fit applied on the all-point amplitude histogram, and the result of the linear fit applied on the current-voltage curve gave a conductance of 3.3 pS, and a reversal potential of +38 mV.

Supplementary Figure S2. Effects of mibefradil on *Am*-Ca_V3, *Am*-Ca_V4 and *Am*-Na_V channels.
a. Full dose-response curves of the effects of Ni^{2+} on *Am*-Ca_V3 Ca^{2+} channel current amplitude. Effect of increasing concentrations of Ni^{2+} on the Ba^{2+} current amplitude recorded on oocytes expressing Am-Ca_V3 for depolarization from 100 mV to -30 mV. The kinetics of the effects of Ni^{2+} on current amplitudes for each concentration is displayed (one data point every 5 s).
b. Kinetics of the effects of mibefradil (10 µM) or its non hydrolysable component, NCC 55-0398 (10 µM), on Ba^{2+} current amplitude recorded during successive depolarizations to -30 mV from a holding potential of -80 mV. Note that in both cases (in the presence of mibefradil or NCC 55-0398) the inhibition was reversible.
c. *Am*-Na_V1 or *Am*-Ca_V4 (4) channels were expressed after injection of *in vitro* synthesized mRNA into *Xenopus* oocytes. Na^+ (Am-Na_V1) or Ba^{2+} (Am-Ca_V4) currents were recorded 2 days later, in voltage-clamp, using step depolarizations in the -120 mV to -10 mV range (+10 mV increment) elicited every 5 s. Mibefradil (10 or 30 µM) was applied directly to the recording solution, using a gravity-driven perfusion, and the effect were quantified as the peak-current amplitude measured in the presence of mibefradil relative to the control current without drug. A bar-graph representing the effects of mibefradil at 10 or 30 µM is shown for oocytes expressing either *Am*-Na_V1 or the *Am*-Ca_V4 channels. No significant effects were recorded (student t-test at 0.05 level).

Supplementary Figure S3: Characterization of potential LVA channels in MBN cells and muscle
a. Left RT-PCR (cropped image) using Am-Ca_V3 specific primers on mRNA isolated from honeybee dissected antenna, leg (metathoracic leg), gut, head or dissected brain. On the left are shown the DNA molecular weights: 250, 500, 700 and 1000 bp. **Right.** RT-PCR (cropped image)for all three types of Ca^{2+} channels using *Am-Ca_V1*, *Am-Ca_V2* and *Am-Ca_V3* specific primers on mRNA isolated from honeybee dissected antennal lobes (AL) or mushroom bodies (MB). On the left are shown the DNA molecular weights: 250, 500, 700 and 1000 bp. Note the poor expression of *Am-Ca_V3* in antennal lobes
b. Left. Cell capacitance (left) for MBN (n=54) neurons and MTib muscle cells (n=32). **Right.** Current activation rate, evaluated as the time necessary to reach the peak-current after the start of the depolarization, was calculated for MBN (n=29) neurons at 0 mV and for Mtib muscle cells at -30 mV and 0 mV (n=17).
c. Left. Reversal potential of Ba^{2+} currents recorded in MBN (MBN, n=24) and muscle cells (MTib) for both HVA and LVA currents, n=32 and 16 respectively. **Right**. R90 values for Ba^{2+} and Ca^{2+} currents calculated from currents recorded MBN neurons and MTib muscle cells (at -30 mV or 0 mV).
d. Left. Superimposed current traces recorded in 10 mM Ca^{2+}- (Ca) and 10 mM Ba^{2+}-containing (Ba) solutions during voltage ramps from -80 mV to 80 mV or +40 mV (duration 150 ms) for MBN (MBN) and muscle cells (MTib). **Right.** Averaged ratios of the current amplitudes recorded in 10 mM Ca^{2+}- or Ba^{2+}-containing solutions for MBN (MBN) or muscle cells (MTib) calculated for depolarisation to 0 mV (HVA) or -30 mV (LVA).
e. Isochronal inactivation curve of Ba^{2+} currents recorded on MBN using a holding potential of -100mV, conditioning depolarizations from -70 to +50 mV and test depolarisation to 0 mV. The Vin was -54 mV.
f. Left Relative fluorescence intensity (ratio 340/380 nm) of Fura2-loaded MBN in response to perfusions of 30 mM KCl (first stimulation) or 30 mM KCl in the presence of 10 µM mbefradil (second stimulation). **Right** Averaged values of pic fluorescence intensities reaches during the KCl perfusion in the two contiditions. Note that mibefradil has no effect on fura2 pic intensity.

Supplementary Figure S4: Effects of mibefradil on memory and locomotion.
a. In the generalization tests, bees from all three groups (control or injected with mibefradil at 0.11 or 1.1 µg/bee) showed a clear generalization gradient, responding differently to the three presented odorants (Cochran's Q test, $Q > 46.7$, $p < 0.001$, 2 df, in all groups). However in the control group, bees responded significantly less to both octanal and 1-hexanol than to the learned odorant, 1-

nonanol (test abc, Mc Nemar test, $p < 0.01$), showing that these bees clearly differentiated between the CS and novel odorants. In both groups injected with Mibefradil, bees responded similarly to 1-nonanol and octanal (Mc Nemar test, $p = 0.095$), and only responded significantly less to 1-hexanol (Mc Nemar test, $p < 0.001$).

b. Exemples of trajectories of 6 bees recorded for 3 min 6 h after injection of 0.11 µg/bee (b-1-3) or 1.1 µg/bee (b-4-6). Positions in the vertical circular arena were detected every s, successive positions linked with doted lines. Control bees injected with vehicle only (not shown, but similar to bee injected with 0.11 µg mibefradil, b1 and b2) tend to explore the space with a preference for positions close to the periphery (thigmotaxis). When injected with 0.11 µg mibefradil, a small number of bees (3/17) stayed in an almost stationary position (B-3). When injected with 1.1 µg mibefradil, the majority of bees (except 3, see b-4 and b-5) had serious locomotion problems, most of them showing almost no displacement (12/15, B-6).

Supplementary Figure S5. Procedure for calculating HVA and LVA peak-currents, half-activation potentials and reversal potentials.

a. Four typical current-voltage curves recorded on individuals muscle cells during voltage-ramps illustrated the variability of the relative expression of LVA and HVA currents in these cells. The currents were normalized to the peak current of the current-voltage curves, and the maximal current densities were 9.1, 7.9, 5.5 and 1.4 nA/nF from left to right.

b. Raw current-voltage curves recorded during voltage ramp from -100 mV to + 40 mV showed a typical hump at -30 mV (Right) indicative of the presence of a LVA Ba^{2+} current. These curves were fitted to an equation representing the sum of two current-voltage curves (see Methods section) to obtain the reversal potential, the half-activation potentials and the curve slopes for the two Ca^{2+} channels components. The peaks of each IV curve were then determined on each individual curve reconstructed using these fitted parameters**.** Averaged current-voltage curves for the two components together (right) and taken individually (left) calculated using either the raw data (for the two component together), of the individual reconstructed curves (for the isolated components).

c. Averaged current-voltage curves for HVA Ba^{2+} currents recorded in these cells (MBN, ALN, MTib and Gt2N) using a step protocol from a holding potential of -100 mV. Note the similar shape.

Supplementary Figure S6: Immunolocalisation of *Am*-Ca_V3 channels on muscle fiber.

a. Confocal image of an isolated leg muscle fibers stained with phalloïdin (Left) and the *Am*-Ca_V3 specific antibody (Right, 1/100 dilution, calibration bar: 30 µm, see method section).

b. Staining quantification was performed by a line scan plot of the fluorescence amplitude of each fluorophore along the fiber axis, and displayed on a X-Y plot. *Am*-Ca_V3 staining was observed in the middle of each phalloïdin band as well as between two successive bands.

c. This distribution was also evidenced by a Fast Fourier Transform analysis of these plots, leading to a calculated ratio of the distance between the middle of two successive phalloïdin bands over the distance between successive *Am*-Ca_V3 staining of 1.6±0.2 (n=14 cells).

Reference List

1. Restituito, S. *et al. J.Neurosci.* **20,** 9046-9052 (2000)

2. Charreton, M. *et al. PLoS.ONE.* **10,** e0144879 (2015)

3. Cens, T. *et al. Insect Biochem.Mol.Biol.* **58,** 12-27

4. Gosselin-Badaroudine P *et al.* Biophysical characterization of the honeybee DSC1 orthologue reveals a novel voltage-dependent Ca^{2+} channel subfamily: Ca_V4. *J.Gen.Physiol.* **148**, 133-145 (2016)

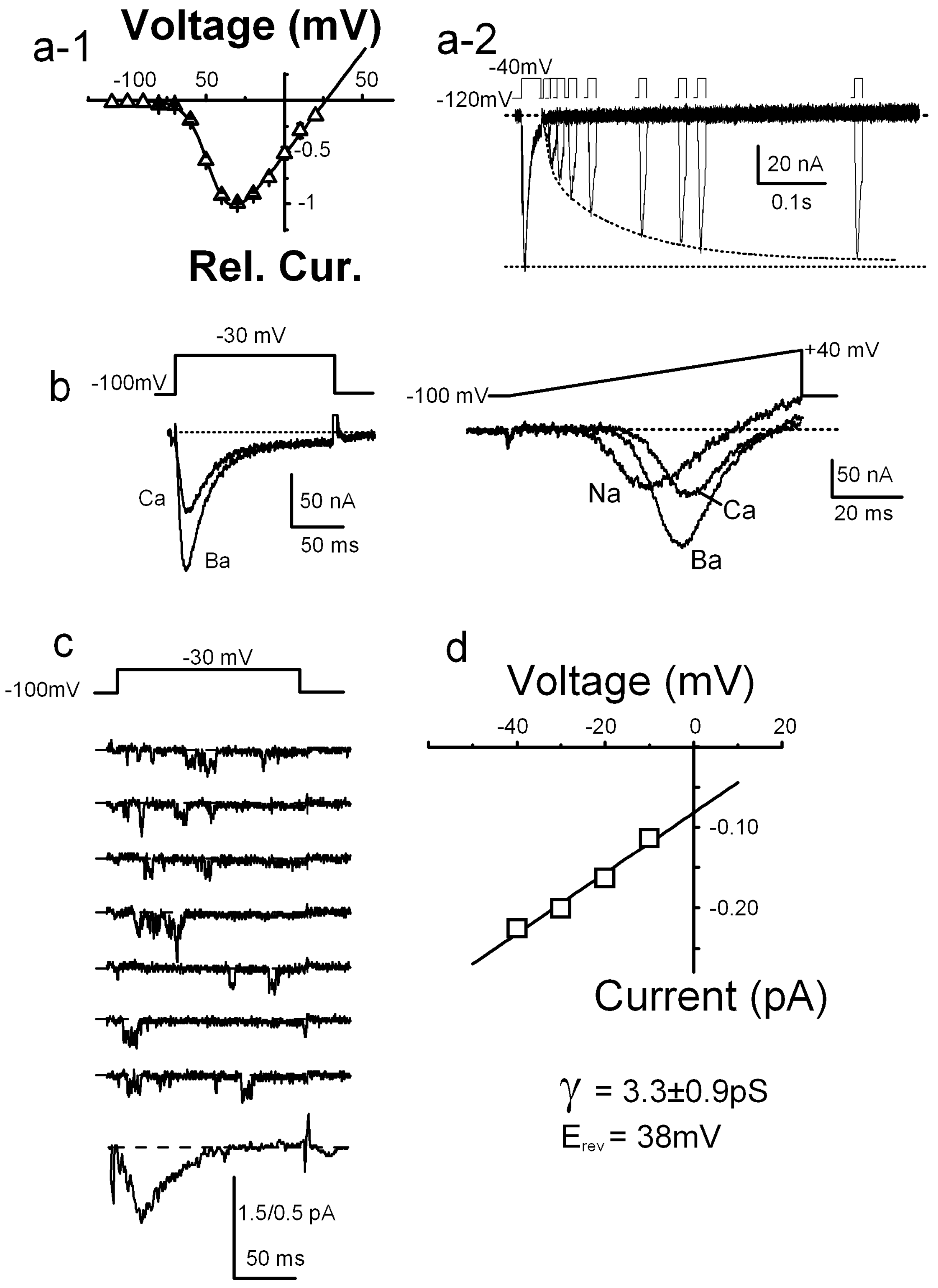
a-1
Voltage (mV)
-100
50
50
-0.5
-1
Rel. Cur.
a-2
-40mV
-120mV
20 nA
0.1s
b
-100mV
-30 mV
Ca
Ba
50 nA
50 ms
-100 mV
+40 mV
Na
Ca
Ba
50 nA
20 ms
c
-100mV
-30 mV
1.5/0.5 pA
50 ms
d
Voltage (mV)
-40
-20
0
20
-0.10
-0.20
Current (pA)
γ = 3.3±0.9pS
Erev = 38mV

Figure S1

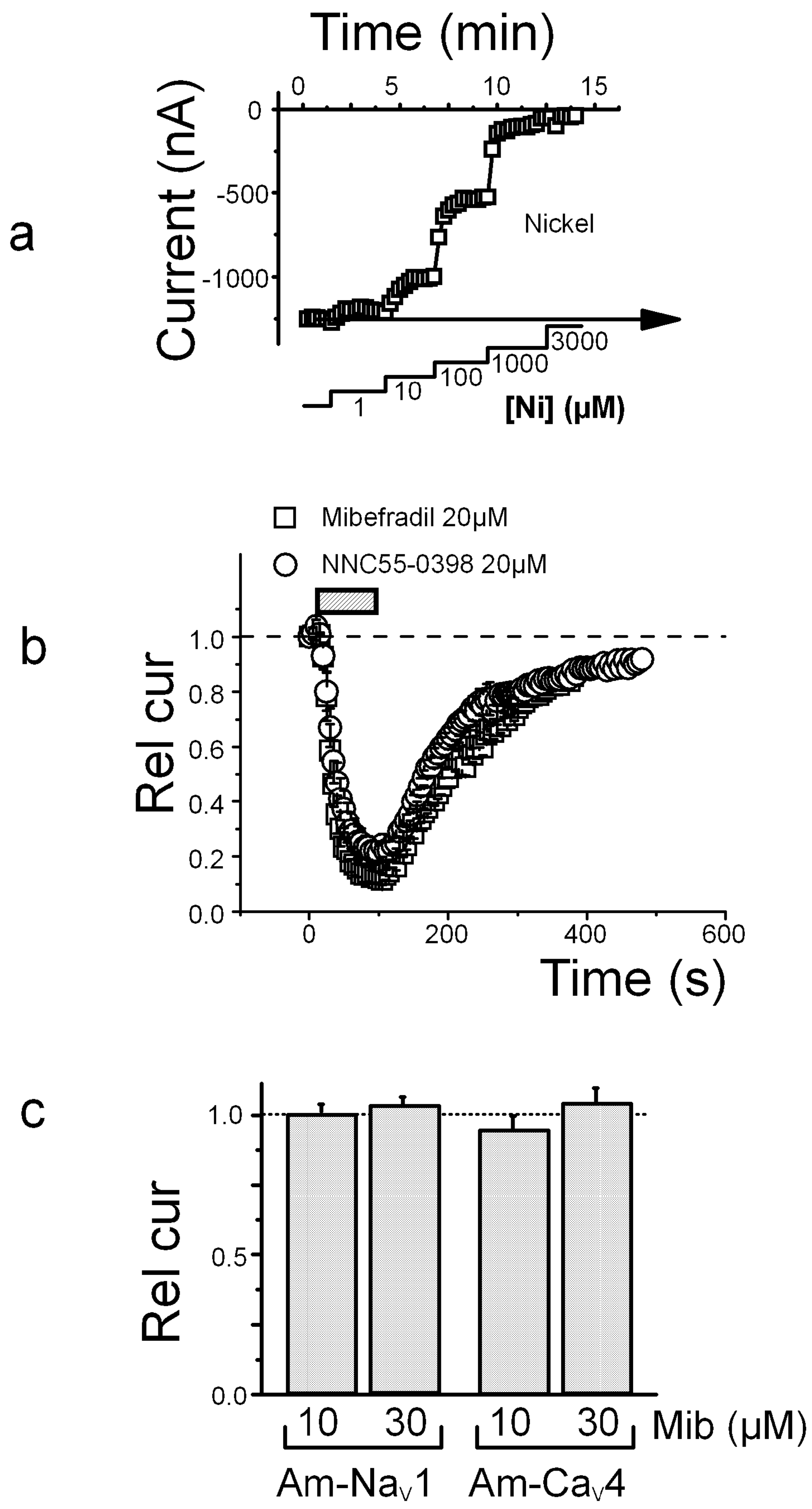

a
Time (min)
0
5
10
15
Current (nA)
0
-500
-1000
Nickel
1
10
100
1000
3000
[Ni] (µM)
b
Mibefradil 20µM
NNC55-0398 20µM
Rel cur
1.0
0.8
0.6
0.4
0.2
0.0
0
200
400
600
Time (s)
c
Rel cur
1.0
0.5
0.0
10
30
10
30
Mib (µM)
Am-NaV1
Am-CaV4

Figure S2

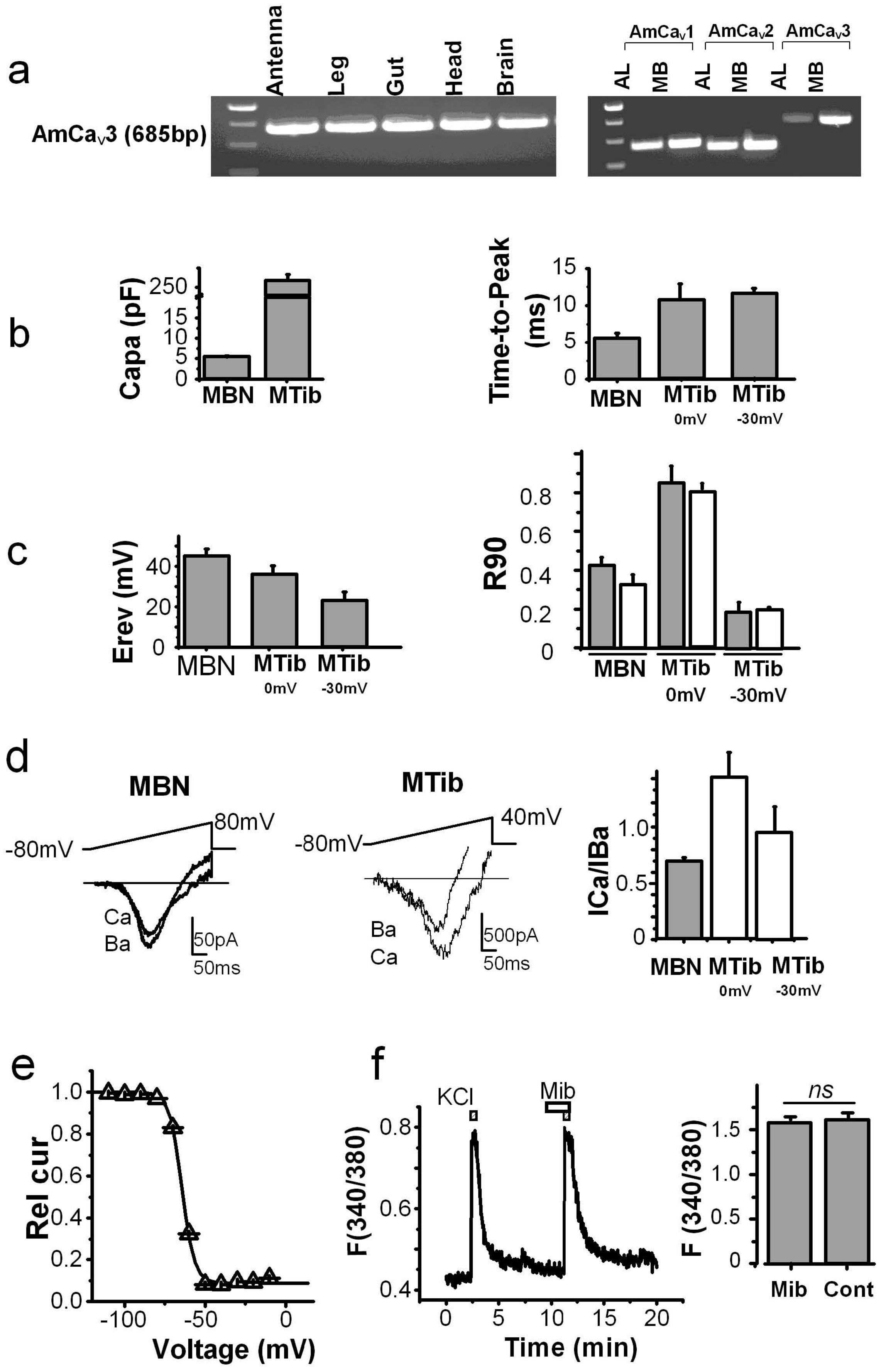

a
Antenna
Leg
Gut
Head
Brain
AmCav3 (685bp)
AmCav1
AmCav2
AmCav3
AL
MB
b
Capa (pF)
MBN
MTib
Time-to-Peak (ms)
0mV
-30mV
c
Erev (mV)
R90
d
MBN
MTib
-80mV
80mV
40mV
Ca
Ba
50pA
500pA
50ms
ICa/IBa
e
Rel cur
Voltage (mV)
f
KCl
Mib
F(340/380)
Time (min)
ns
Cont

Figure S3

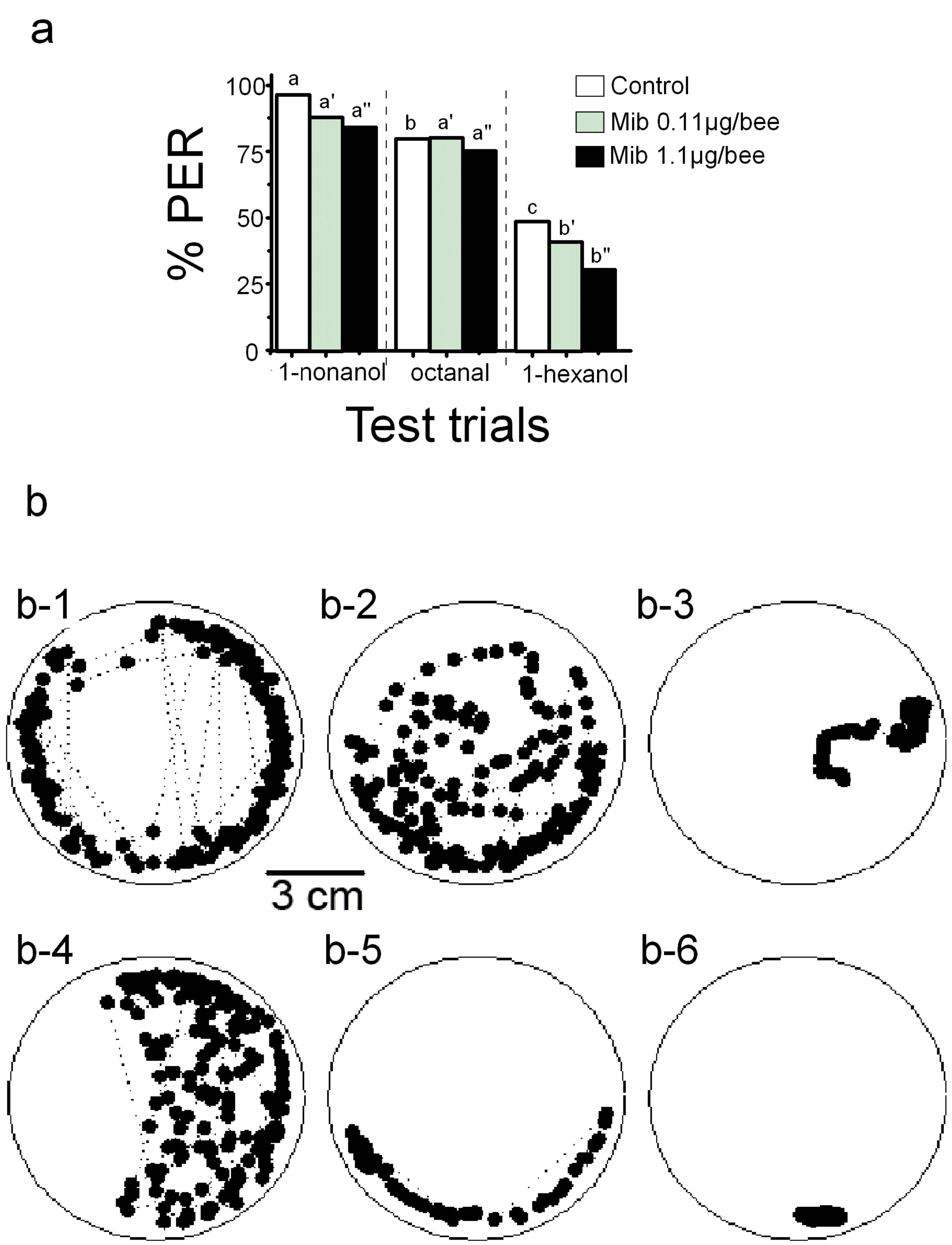
a
% PER
100
75
50
25
0
a
a'
a''
b
a'
a''
c
b'
b''
1-nonanol
octanal
1-hexanol
Test trials
Control
Mib 0.11µg/bee
Mib 1.1µg/bee
b
b-1
b-2
b-3
b-4
b-5
b-6
3 cm

Figure S4

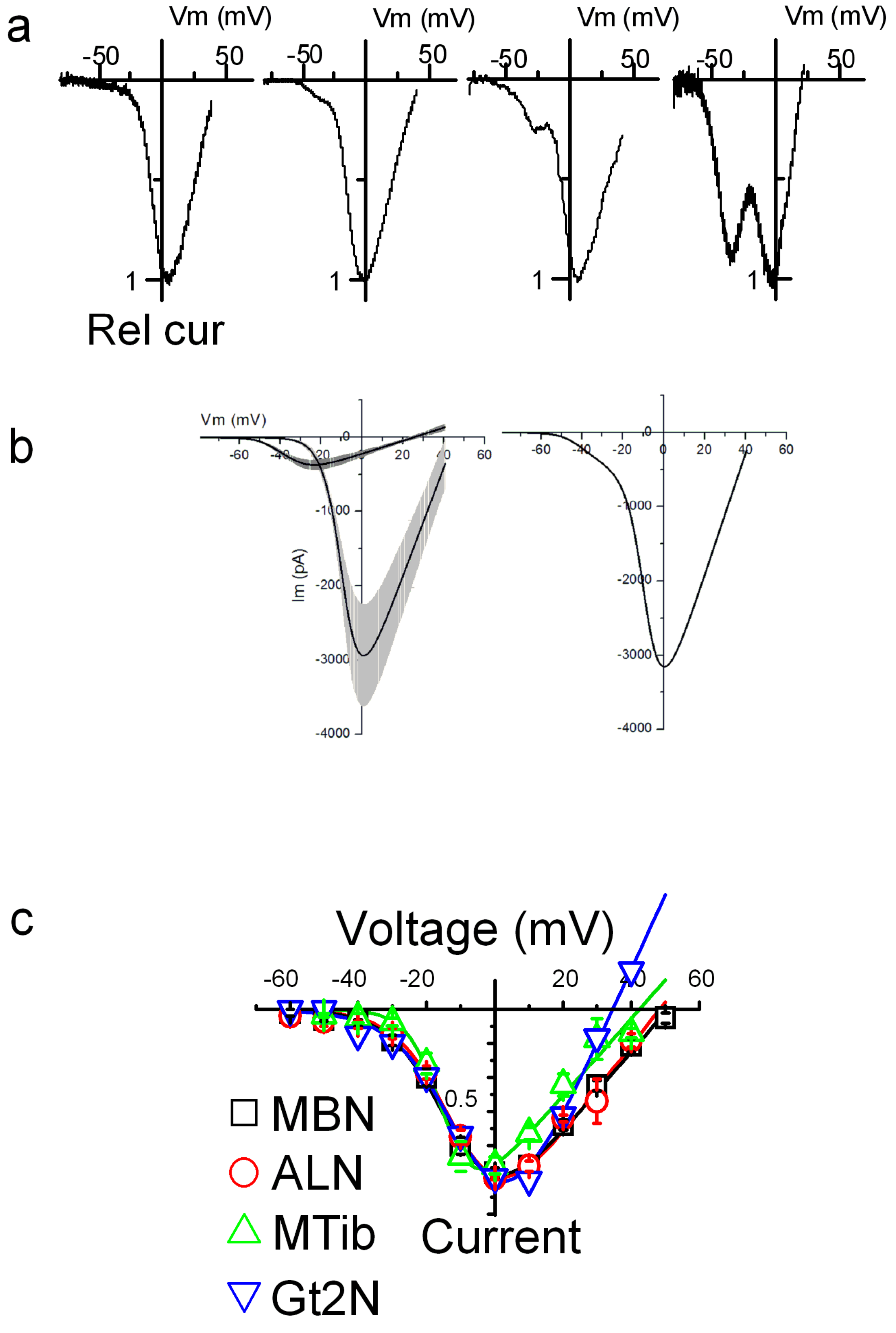
a
Vm (mV)
-50
50
1
Rel cur
b
Vm (mV)
Im (pA)
-4000
c
Voltage (mV)
-60
-40
-20
20
40
60
0.5
MBN
ALN
MTib
Gt2N
Current

Figure S5

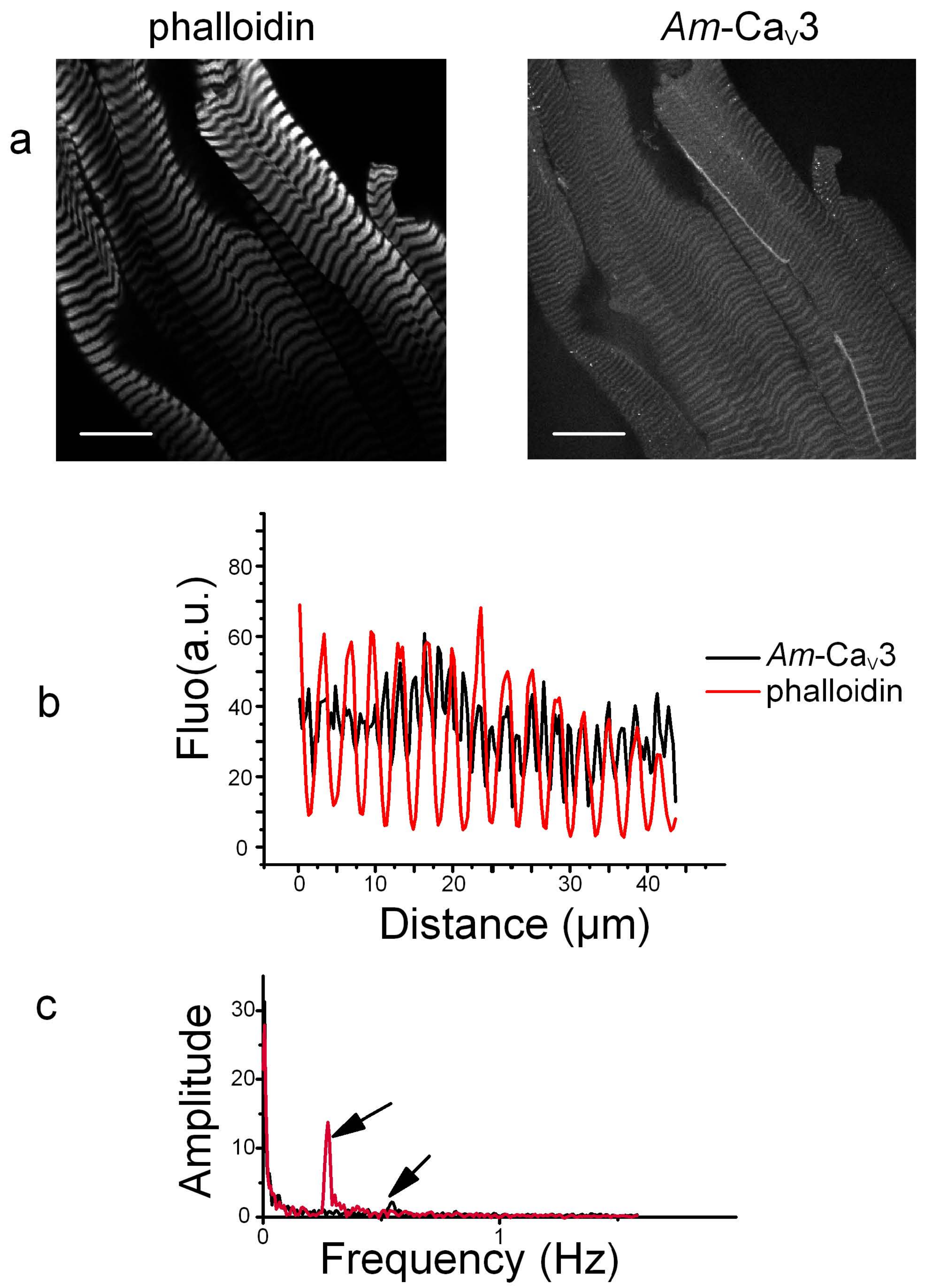

phalloidin
Am-Ca$_V$3
a
b
c
Fluo(a.u.)
Distance (µm)
Am-Ca$_V$3
phalloidin
Amplitude
Frequency (Hz)

Figure S6
